# Homeostasis of Gut Microbiota Protects against Susceptibility to Fungal Pneumonia

**DOI:** 10.1002/advs.202416455

**Published:** 2025-08-04

**Authors:** Jian Ji, Yongli Ye, Jiadi Sun, Lina Sheng, Jinyou Li, Jin Yang, Bing Wu, Yuting Wang, Xingxing Gao, Liang Luo, Jianfeng Ping, Yinzhi Zhang, Xiulan Sun

**Affiliations:** ^1^ School of Food Science and Technology, International Joint Laboratory on Food Safety, Synergetic Innovation Center of Food Safety and Quality Control Jiangnan University Wuxi Jiangsu 214122 P. R. China; ^2^ Key Laboratory of Screening, Prevention, and Control of Food Safety Risks State Administration for Market Regulation Beijing 100176 P. R. China; ^3^ Affiliated Hospital of Jiangnan University Wuxi Jiangsu 214122 P. R. China; ^4^ Department of Critical Care Medicine Wuxi No.2 People's Hospital Wuxi Jiangsu 214002 P. R. China; ^5^ Laboratory of Agricultural Information Intelligent Sensing, School of Biosystems Engineering and Food Science Zhejiang University Hangzhou 310058 P. R. China; ^6^ Innovation Platform of Micro/Nano Technology for Biosensing, ZJU‐Hangzhou Global Scientific and Technological Innovation Center Zhejiang University Hangzhou 311200 P. R. China; ^7^ Analysis and Testing Center Jiangnan University Wuxi Jiangsu 214122 P. R. China; ^8^ Institute of Future Food Technology JITRI Yixing 214200 P. R. China

**Keywords:** fungal pneumonia, fusarium graminearum, gut‐lung axis, lung metabolism, lung microbiota

## Abstract

Fungal pneumonia is a serious disease with great harm and high prevalence, presenting significant challenges in diagnosis and treatment. The gut and respiratory microbiota play a critical role in protecting lung health against fungal pneumonia. Here, it is established fungal pneumonia by infection via the sinopulmonary route with *Fusarium graminearum (F. graminearum)* to investigate the influence of gut microbiota state on susceptibility to fungal pneumonia in BALB/c mice. This findings revealed that *F. graminearum* spore exposure not only impaired pulmonary clearance mechanisms but also significantly upregulated the expression of proinflammatory cytokines, including interleukin‐6 (IL‐6), interleukin‐1β (IL‐1β), and tumor necrosis factor‐α (TNF‐α). Moreover, spore invasion led to an increase in *Staphylococcus* abundance and activation of both triglyceride and galactose metabolic pathways. Antibiotic treatment disrupted the gut and respiratory microbiota, facilitating *F. graminearum* lung colonization, which is evidenced by elevated inflammatory markers in alveolar fluid and dysregulated lung metabolism. It is demonstrated that the gut microbiota influences susceptibility to fungal pneumonia by acting as an intermediary in the gut‐lung axis through the bloodstream, thereby modulating lung metabolism and inflammatory responses. These findings open avenues for novel therapeutic strategies, such as gut microbiota modulation, for the prevention and treatment of fungal pneumonia.

## Graphical abstract

Fungal pneumonia induces inflammation, shown by heightened IL‐6, IL‐1β, TNF‐α levels and a growth in *Staphylococcus* in the alveolar flora. The gut microbiota, acting through the gut‐lung axis via blood, impacts fungal pneumonia susceptibility by altering lung metabolism and inflammatory responses.

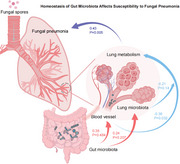

## 1 Introduction

Pneumonia in general is widespread, with millions of cases reported worldwide each year.^[^ ^1^ ^]^ Pneumonia is a common and serious respiratory infection that can be classified into community‐acquired pneumonia (CAP) and hospital‐acquired pneumonia (HAP). Within CAP, ≈5%–15% of cases are classified as aspiration pneumonia.^[^ ^2^ ^]^ The air is laden with pollen, fungal spores, viruses, and bacteria; these airborne pollutants can enter the human body via the respiratory system, increasing the incidence of chronic non‐communicable lung diseases, or pneumonia.^[^ ^3^ ^]^ Fungal pneumonia specifically poses great harm. It can be difficult to diagnose and treat, leading to prolonged illness and a higher risk of complications.^[^ ^4^ ^]^ Studies have shown that the prevalence of fungal pneumonia is increasing, especially in immunocompromised patients. Emerging fungal pneumonia infections have emerged due to population and environmental changes. Fungi can cause a range of opportunistic infections in humans, ranging from inhalation infection to disseminated infections, depending on the immune status of the lung.^[^ ^5^ ^]^ Particularly in the post‐pandemic era, evidence suggests that while most individuals infected with COVID‐19 fully recover, 10%–20% experience various intermediate and long‐term effects after recovering from the initial disease^[^ ^6^ ^]^

In the intricate environment of lung well‐being, the ebb and flow of lung metabolic intricacies, shaped by a harmonious interplay between lung microbiota and environmental stimuli—most notably, fungal spores—play a critical role.^[^ ^7^ ^]^ These metabolic perturbations, capable of initiating inflammatory responses across the pulmonary landscape, may disrupt microbial harmony, provoke immune responses, and hint at emerging pathological states.^[^ ^8^, ^9^ ^]^ The invasion of fungal spores into the respiratory system triggers a cascade of events, starting with the activation of macrophagic inflammasomes by galactosaminogalactan (GAG) on the spore surface, which signals the onset of protective immunity.^[^ ^10^ ^]^ Additionally, the strategic removal of melanin from the spore's surface not only stimulates glycolytic pathways but also intersects with the regulation of intracellular calcium ion dynamics.^[^ ^9^ ^]^ The entry of fungal spores leads to a marked release of lactate dehydrogenase (LDH) in A549 cells and the concurrent detachment of these pulmonary epithelial cells,^[^ ^11^ ^]^ setting the stage for Th‐2 mediated inflammatory responses and the subsequent development of allergic reactions.^[^ ^12^ ^]^ Alveolar macrophages play a pivotal role in this scenario by phagocytizing fungal spores and igniting inflammatory responses.^[^ ^13^ ^]^ Exposure to volatile compounds from *Aspergillus fumigatus* disturbs the cytokine equilibrium, notably affecting IL‐6 and IFN‐γ levels.^[^ ^14^ ^]^ Such fungal interactions also facilitate the emergence of adaptive immune responses, generating antigen‐specific T helper (Th) effector cells, regulatory T cells (TReg), and B cells, with CD4+CD25+ TReg cells releasing IL‐10 and TGF‐β to prevent the complete clearance of the fungal presence.^[^ ^15^ ^]^

The homeostasis of the gut microbiota is crucial for the body, which has a significant influence on shaping both local and systemic immune defenses.^[^ ^16^ ^]^ Recent studies have increasingly highlighted the significant influence of gut microbiota and its metabolic derivatives on shaping both local and systemic immune defenses. This research has led to the identification of the “gut‐lung axis,” a concept that underscores the interconnection between the gastrointestinal tract and the lungs, playing a crucial role in maintaining metabolic balance and influencing the development of pulmonary diseases.^[^ ^17^, ^18^, ^19^, ^20^ ^]^ Airborne particulates, comprising various toxins and metals, can induce an imbalance in the lung and gut microbial consortia, thereby compromising lung function.^[^ ^21^ ^]^ The gut‐lung axis, thus, represents a critical biological conduit for understanding pulmonary disease mechanisms. A diverse microbiota is beneficial for host defense; however, antibiotic use can alter the gut microbiome, indirectly leading to systemic changes.^[^ ^22^, ^23^, ^24^ ^]^ The easy availability of antibiotics and misconceptions about their use have resulted in overuse and misuse. It has been reported that only 10% of upper respiratory tract infection cases are caused by bacteria that warrant antibiotic treatment, while the remaining 90% are viral in origin. Long‐term antibiotic treatment in tuberculosis patients has been associated with cognitive impairment and delusional thinking.^[^ ^25^ ^]^ Furthermore, antibiotic use may disrupt the intestinal epithelial barrier, increasing susceptibility to infections from Salmonella, Shigella, and Clostridium difficile.^[^ ^26^ ^]^ Alterations in gut microbiota, through systemic circulation, can impact lung metabolism and, consequently, respiratory health.^[^ ^27^, ^28^ ^]^ This intricate dialogue underscores the significance of gut microbiome in preserving lung health, as improving the status of gut microbiota can regulate pulmonary immunity through the gut‐lung axis.^[^ ^29^, ^30^ ^]^

We form fungal pneumonia by infecting through the sinopulmonary route with *Fusarium* to study the impact of the state of the gut microbiota on the susceptibility to fungal pneumonia. By employing a mouse model of fungal spore exposure, we conducted a comprehensive analysis of its direct impact on lung metabolism and the lung's microbiome. Additionally, through antibiotic pre‐treatment, we explored the gut microbiota's regulatory influence on fungal pneumonia.

## 2 Results

### 2.1 Lung Infection by *F. Graminearum* on Alveolar Inflammation and Microbiota

**Figure 1:**
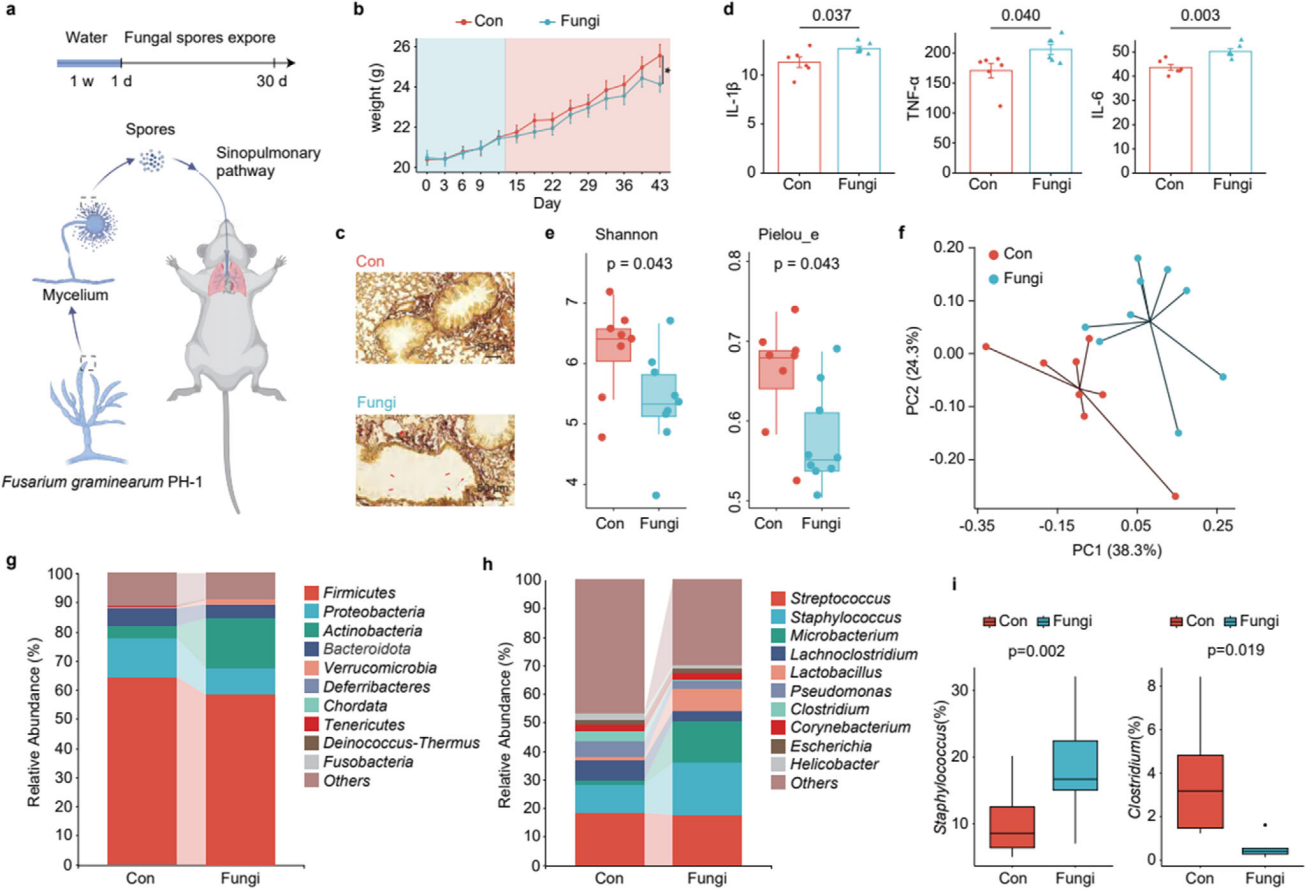
Impact of lung *F. graminearum* infection on alveolar inflammation and microbiota in mice. a) schematic of experimental workflow; b) body weight variation (n = 20); c) lung tissue Periodic acid‐silver methenamine staining with fungal spores marked by red arrow; d) changes in inflammatory cytokines IL‐6, TNF‐α, IL‐1β in mouse BALF due to fungal spore exposure (n = 6); e) α‐diversity indices of mice BALF bacterial communities (n = 9); f) PCA of mouse BALF bacterial communities (n = 9); g) phylum‐level bacterial abundance bar graph in mouse BALF; h) genus‐level bacterial abundance bar graph in mouse BALF; i) abundance box plots of *Staphylococcus* and *Clostridium*.

The *F. graminearum* PH‐1 spores were isolated using a 0.05% Tween‐80 phosphate‐buffered saline solution, followed by a meticulous washing and centrifugation process to procure a spore suspension, the concentration of which was accurately determined through microscopic enumeration. Subsequently, these spores were intratracheally administered into BALB/c mice, thereby simulating an exposure scenario. After a two‐week acclimatization period, the mice commenced a regimen of bi‐weekly spore exposure treatments (**Figure** **1**a). During the 30‐day exposure window, we noted a significant decrease in the body weight of the spore‐exposed mice (Fungi group) in comparison to their control counterparts (Figure 1b, Table S1, Supporting Information). No obvious differences were observed in the mice's general behavior, including activity levels and feeding habits. The lungs of both the control and spore‐exposed groups were subjected to Periodic acid‐silver methenamine (PASM) staining, which predominantly marks fungal polysaccharides within the cell walls, resulting in a black coloration. The control group's lung sections revealed no significant presence of fungi, whereas in the Fungi group, structures resembling the rod‐shaped spores of *F. graminearum* PH‐1 were observed (Figure 1c). Upon dissection, comparison of lung images of infected and normal mice revealed hemorrhagic areas in the infected mice's lung tissues (Figure S1, Supporting Information). Additionally, samples extracted from the bronchoalveolar lavage fluid (BALF) of these mice were analyzed for inflammatory cytokines using an ELISA test. The results indicated a marked increase in the levels of IL‐6, IL‐1β, and TNF‐α (*p* < 0.05), as shown in Figure 1d. Through centrifugation of BALF and subsequent sequencing of the microbial populations (Table S2, Supporting Information), 16S rRNA analysis revealed that spore exposure led to a decline in lung microbial diversity (Shannon index) and evenness (Pielou's e index), as evidenced by a p‐value less than 0.05 in Figure 1e. Utilizing principal component analysis (PCA) at the operational taxonomic unit (OTU) level, we demonstrated notable differences in beta‐diversity between the control and Fungi groups, indicating a clear separation as shown in Figure 1f. Cluster analysis further confirmed the significant impact of *F. graminearum* on the composition of the lung microbiota in mice. A deeper investigation into the microbial composition revealed that at the phylum level, the abundance of Firmicutes, Proteobacteria, and Bacteroidota decreased, while that of Actinobacteria and Verrucomicrobia increased (Figure 1g). Several studies have identified differences in the gut microbiota of *Firmicutes*, *Staphylococcus* in asthma patients.^[^ ^31^ ^]^ Some authors have also proposed the involvement of *Firmicutes* in the gut ecological dysregulation of asthma‐COPD overlap syndrome mice.^[^ ^32^ ^]^ In addition to *Firmicutes*, *Staphylococcus*, it was found that PM exposure affects the abundance of *Actinobacteria*.^[^ ^33^ ^]^ By comparing the intestinal flora of the Con and Fungi groups, it was found that there were significant differences in these bacteria in both groups as well. At the genus level, the abundance of *Lachnoclostridium* and *Clostridium* was reduced, whereas *Staphylococcus*, *Microbacterium*, and *Lactobacillus* increased (Figure 1h). Notably, Figure 1i highlighted a significant upregulation of *Staphylococcus* (*p* = 0.002) and a notable downregulation of *Clostridium* (*p* = 0.019). A significant increase of *Staphylococcus* has been pointed out in previous studies as a potential cause of secondary pneumonia in the altered lung microenvironment following influenza infection.^[^ ^34^ ^]^ *Clostridium* plays an important role as a beneficial bacterium in the fight against influenza viral pneumonia,^[^ ^35^ ^]^ in immunotherapy for lung cancer,^[^ ^36^, ^37^ ^]^ and in neural regeneration.^[^ ^38^ ^]^ Significant down‐regulation of *Clostridium* may interfere with the pulmonary immune response, thereby exacerbating the inflammatory response in the lungs. Previous studies on *Lactobacillus*‐associated pneumonia found it usually has mild pathological features like patchy alveolar inflammation, a Th2‐biased immune response, and the bacteria mainly colonize in the upper respiratory tract mucosa. In our study, *F. graminearum*‐induced pneumonia in mice shows severe tissue damage, diffuse alveolar thickening, a strong pro‐inflammatory Th1‐type immune response, and extensive fungal hyphae infiltration in the lung parenchyma. Regarding the changes in the lung microbiota, we propose that this is more likely a secondary response of the lung microbiota to the initial infection by *F. graminearum* rather than a causative factor of pneumonia.

### 2.2 Impact of *F. Graminearum* Colonization on Lung Metabolism and Pneumonia

**Figure 2:**
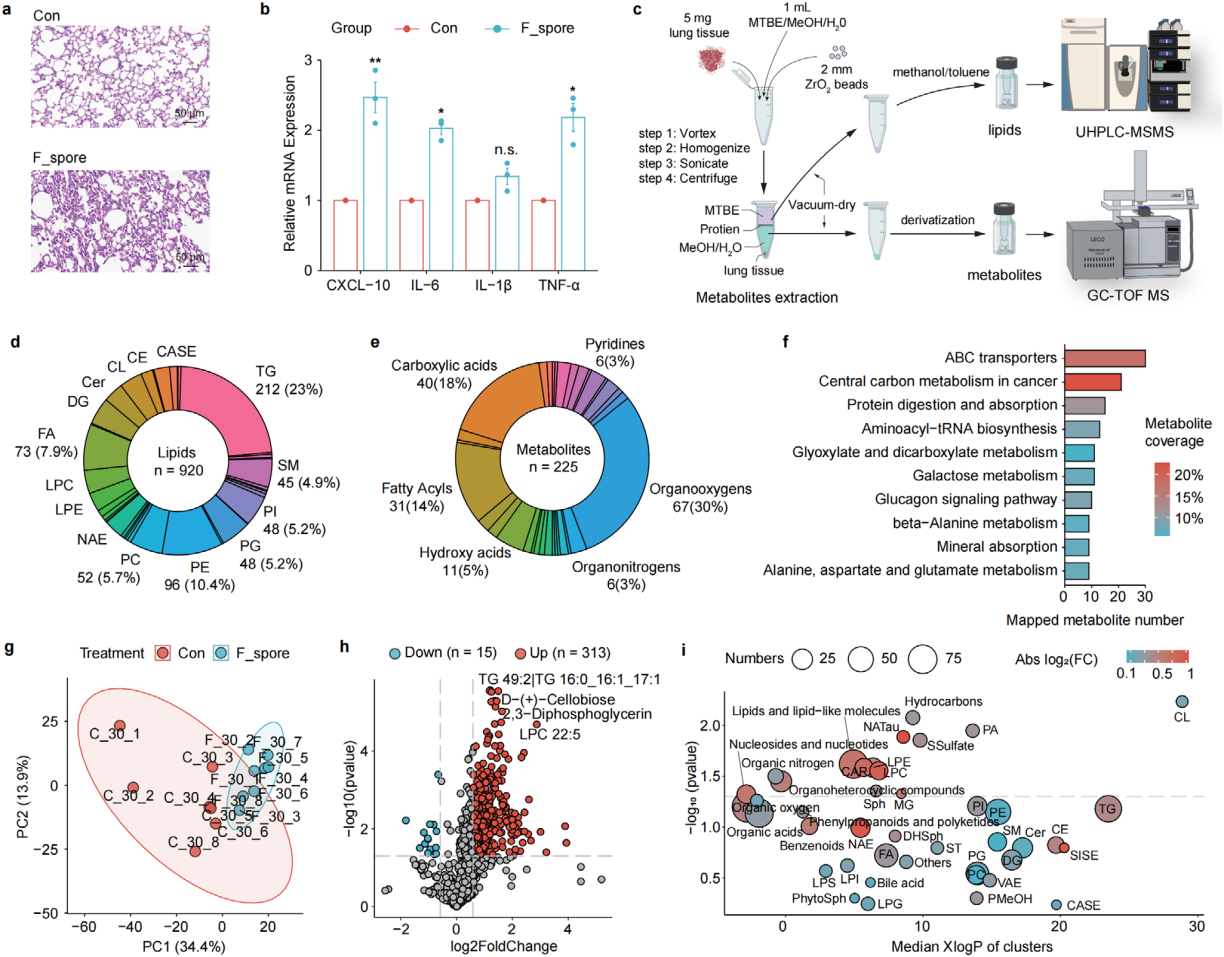
Pneumonia and lung metabolism caused by the colonization of *F. graminearum*. a) The H&E staining of lung tissue; b) changes in lung tissue mRNA expression; c) schematic of lung tissue metabolite extraction and analysis; d) identification of non‐polar metabolites (lipid metabolites); e) identification of polar metabolites; f) metabolic pathways involving identified polar metabolites; g) PCA of all metabolites; h) volcano plot of all metabolites; i) ChemRICH clustering of 1145 altered lung metabolites by chemical similarity with axes of mediation logarithmic octanol‐water partition coefficients (XlogP) and significance based on the Kolmogorov–Smirnov test; node size indicates compound numbers per cluster set, and node color scales the proportion of Con‐enriched versus Fungi enriched metabolites. Data are mean ± SEM. * and ** denote *p*‐values < 0.05 and 0.01, respectively.

Subsequent to fungal spore administration, we subjected murine lung tissues to hematoxylin and eosin (H&E) staining, revealing pronounced alveolar septal thickening, robust inflammatory cell infiltration, including neutrophils, macrophages and lymphocytes, and significant alveolar fusion and fibrosis (**Figure** **2**a). This was corroborated by elevated expression of inflammatory chemokine CXCL‐10, along with cytokines IL‐6, IL‐1β, and TNF‐α in the spore‐exposed group, affirming inflammatory onset (*p* < 0.05, Figure 2b). These data are sufficient to prove that fungal pneumonia has been developed in the lungs of mice.

Metabolomic dissection of lung tissues discerned 920 lipids (Table S3, Supporting Information) and 225 polar metabolites (Table S4, Supporting Information) via liquid chromatography‐mass spectrometry (LC‐MS) and gas chromatography‐time of flight mass spectrometry (GC‐TOF‐MS), as demonstrated in Figure 2c. Notably, spore exposure markedly altered triglyceride (TG) metabolism, evidenced by the identification of 212 TGs, constituting ≈23% of lipids detected (Figure 2d). Polar metabolites comprised organooxides, carboxylic acids, and acyls at proportions of 30%, 18%, and 14%, respectively (Figure 2e). Pathway analysis of all metabolites highlighted enrichment in ABC transporters, central carbon metabolism in cancer, and galactose metabolism (Figure 2f).

PCA clarifies the variations in lung metabolism resulting from exposure to fungi compared to no exposure (Figure 2g). Additionally, a volcano plot illustrates the specific changes in metabolites, with 313 substances being upregulated and 15 downregulated (Figure 2h). A comprehensive landscape view was obtained through Chemical Similarity Enrichment Analysis (ChemRICH), clustering forty chemical categories spanning a wide lipophilicity spectrum, with variations in compound quantity and overall trends indicated by node colors; red nodes denoted a greater quantity and/or more significant overall change of metabolites enriched in the Fungi group compared to Con group (Figure 2i). Detailed categorization of metabolites with significant quantity and variation was conducted, with non‐polar metabolites such as CAR/LPC/TG and sphingomyelins (SM) showing significant changes. TGs, a major form of energy storage, exhibited significant differential changes upon spore exposure. Their abnormal metabolism may lead to impaired immune cell function. It has been shown that accumulation of neutral lipids (triglycerides) promotes tumor cell colonization and metastasis in the lungs,^[^ ^39^, ^40^ ^]^ so it is speculated that triglycerides also affect the nutritional environment of the fungus and thus should influence the infection. It has also been shown that elevated triglyceride levels are associated with suppression of immune cell function, which may reduce host clearance of fungi.^[^ ^41^ ^]^ Among polar metabolites, lipids and lipid‐like molecules, heterocyclic organic compounds, organooxides, nucleosides, nucleotides, and their analogs showed substantial alterations, particularly carbohydrates within organic oxides, which were markedly upregulated, as annotated in Figure S2 (Supporting Information). These findings underscore the significant impact of fungal spore exposure on murine lung metabolism, with a pronounced effect on lipid metabolism.

**Figure 3:**
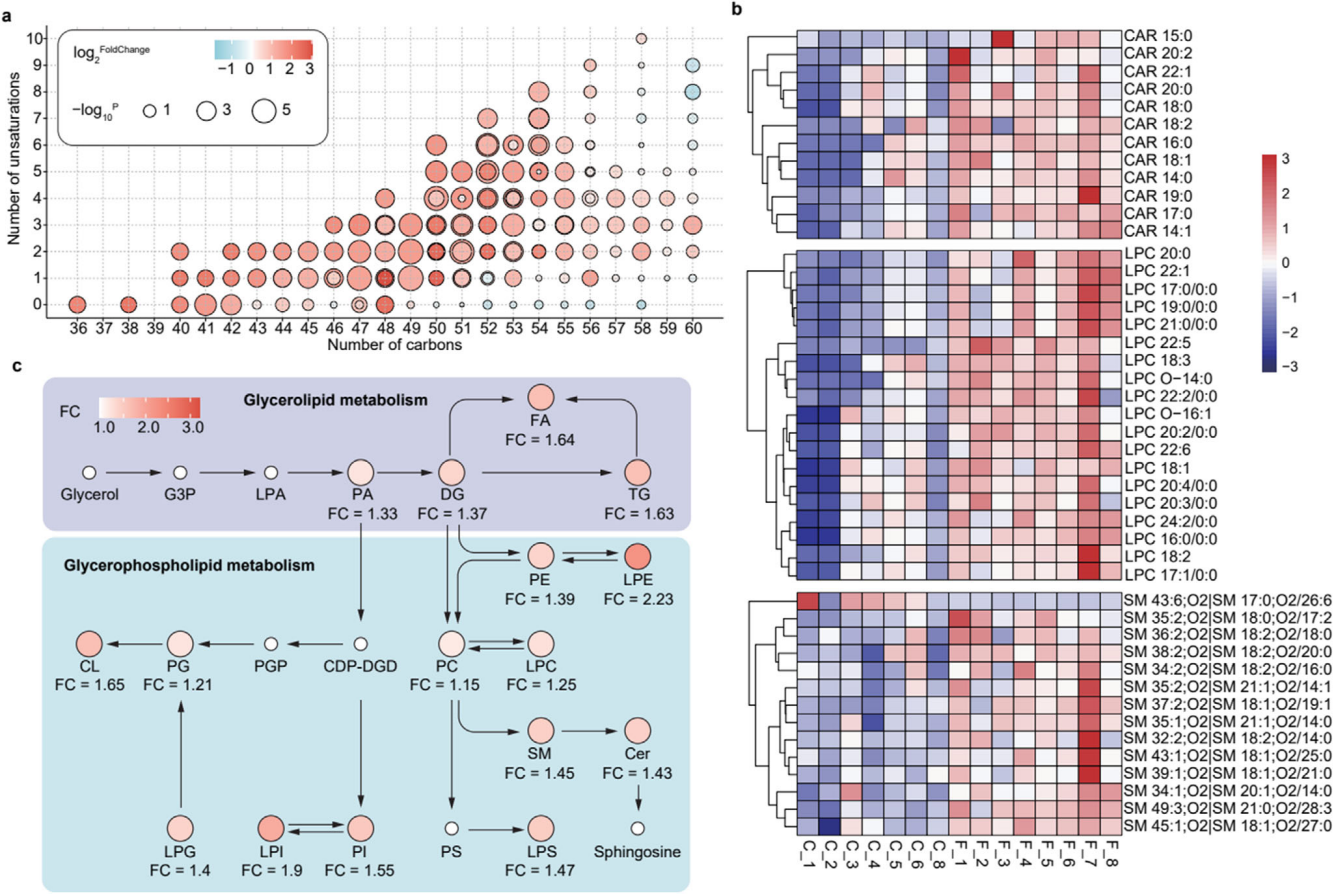
Effect of fungal pneumonia infection on lipid metabolism in mice lungs. a) Bubble plots are utilized to exhibit the carbon number and the count of unsaturated bonds in TG, illustrating the variances with and without spore exposure, each point represents a different lipid molecule, with differences enriched by carbon number and unsaturation degree; b) heatmap of CAR, LPC, SM indicating downregulation (blue) and upregulation (red); c) the color intensity of lipids in the glycerolipid and glycerophospholipid metabolic pathways signifies the differences with and without exposure to *F. graminearum*.

A detailed analysis of lipids was also encompassed in our study, with a particular focus on the top 4 lipids of TGs, cholesteryl esters (CAR), lysophosphatidylcholines (LPC), and SM. The differential expression of TGs was further categorized based on unsaturation levels and carbon chain lengths (**Figure** **3**a), revealing significant upregulation in most TGs, except for those with higher carbon numbers or no unsaturated bonds. The pronounced increase in unsaturated TGs, containing one or more unsaturated fatty acids, underscores their critical role in energy balance and biological signaling.^[^ ^42^ ^]^ The number of carbon atoms and unsaturated bonds of LPC, CAR, and SM are shown in the attached Figure S2 (Supporting Information). This phase involved the comparative heatmap visualization of CAR, LPC, and SM, all of which exhibited an overall upregulation trend (Figure 3b). The elevated levels of CAR, a cholesterol‐fatty acid ester, could potentially impact cardiovascular health.^[^ ^43^ ^]^ LPCs, crucial phospholipids, play a vital role in cell membrane integrity and cellular signaling,^[^ ^44^ ^]^ while SMs, key components of cell membranes, are involved in regulating cell proliferation, differentiation, and apoptosis.^[^ ^45^ ^]^ Pathway enrichment analysis highlighted glycerolipid and glycerophospholipid metabolism as the most impacted pathways in non‐polar metabolites (Figure 3c), with pathway maps annotated with fold‐change values of differential metabolites, visually representing the spore exposure's impact on these pathways. The abnormal metabolism of TG seriously affects the normal energy metabolism of the lungs. TG breakdown in bronchial epithelial cells is mediated by PPARα, which activates mitochondrial fatty acid β‐oxidation, and PPARγ, stimulating adipogenesis.^[^ ^46^ ^]^ This metabolic process is essential for supplying energy‐rich fatty acids, supporting cellular energy homeostasis and structural integrity. Activation of PPARα also promotes mitochondrial biogenesis, enhancing metabolic efficiency.^[^ ^47^, ^48^ ^]^

**Figure 4:**
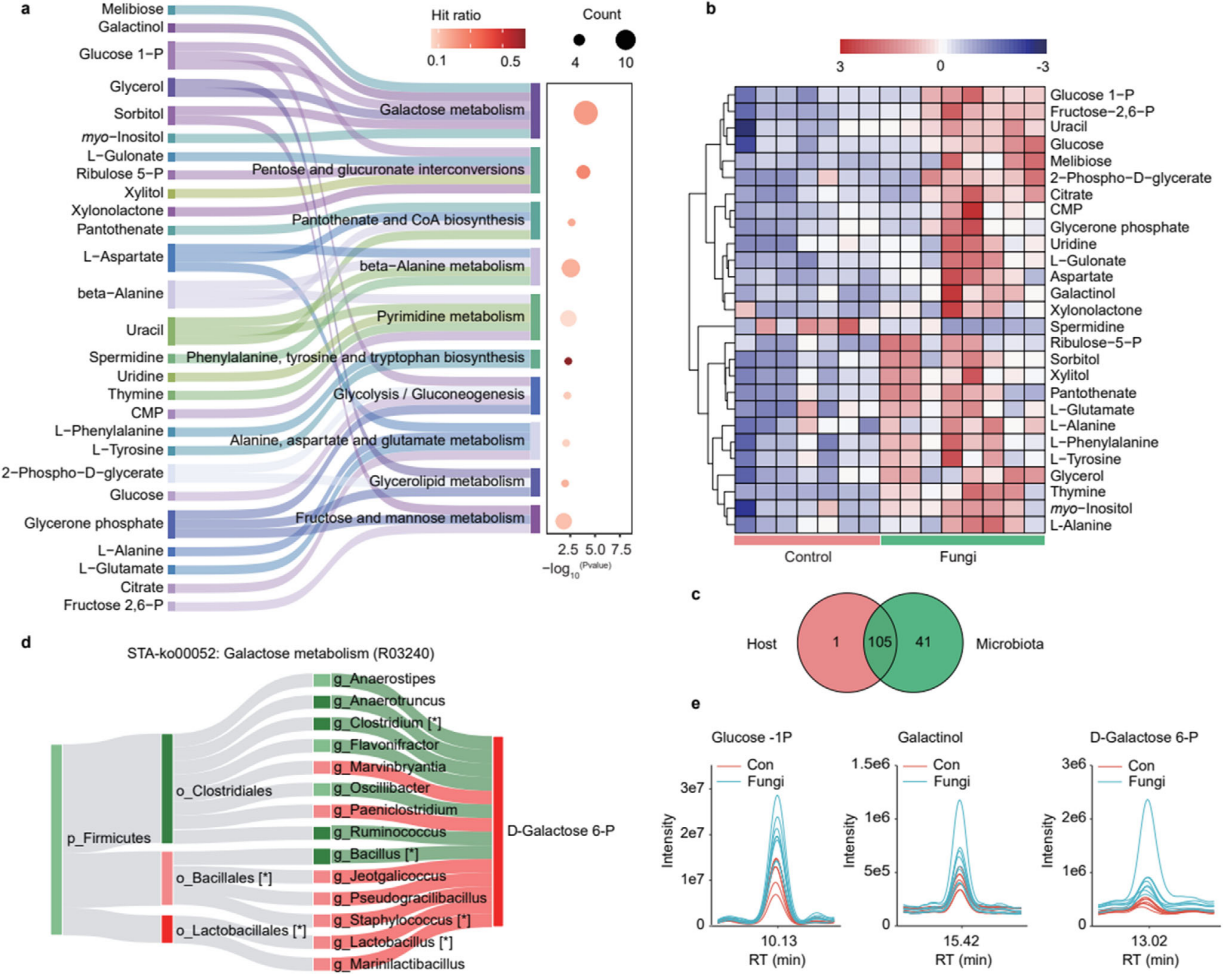
Effect of fungal pneumonia infection on primary metabolism in mice lungs. a) Integrate a Sankey diagram with a scatter plot to visualize the pathway enrichment of statistically different polar metabolites; b) heatmap of the top 20 differential metabolites, with blue and red indicating downregulation and upregulation, respectively; c) Venn diagram of metabolites in Host and microbial communities; d) STA‐Sankey network of the galactose metabolism pathway (R00052) with asterisks (*) denoting statistically significant correlations with metabolites; red (or green) nodes for up‐regulation (or down‐regulation); red (or green) bands for positive (or negative) correlations with metabolites; deep red/green for *p* < 0.05; e) mass spectral abundance charts of galactose metabolism‐related metabolites glucose‐1‐P, galactinol, D‐galactose‐6‐P.

Beyond non‐polar metabolite analysis, this study probed into the significantly altered polar metabolites via pathway enrichment analysis. By incorporating differential metabolites into the KEGG database, a predominant enrichment was observed in the galactose metabolism pathway (**Figure** **4**a). Galactose is an important precursor substance for fungal cell wall synthesis, therefore abnormal galactose metabolism in the organism may affect the nutrition, growth and colonization required by fungi.^[^ ^49^ ^]^ It has been found that galactose lectin‐3 (Galectin‐3) expression is up‐regulated in pulmonary fibrosis, which affects the pathogenicity of fungi by regulating galactose metabolism. In addition to this, fungi may evade immune surveillance by modulating galactose metabolism in host cells. For example, Candida albicans may avoid recognition by phagocytes by reducing cell wall galactose exposure within the host.^[^ ^50^ ^]^ When sorted based on its significance, hit ratio, and the count of involved differential metabolites, besides galactose metabolism (melibiose, galactinol, glucose 1‐P, glycerol, sorbitol, and *myo*‐inositol) ranking first, the pentose and glucuronate interconversions pathway (glucose‐1‐P, L−gulonate, ribulose‐5‐P, xylitol, and xylonolactone) is enriched as the second‐ranked pathway. This can be preliminarily understood as, after settling in the lungs of mice, *F. graminearum* not only affect the pulmonary inflammation and lung tissue TG metabolism mentioned earlier but also influence the metabolism of lung pentoses and hexoses. In addition, among the top 10 enriched pathways, we also observed glycolysis/gluconeogenesis, glycerolipid metabolism, and fructose and mannose metabolism, all of which are related to sugar metabolism, which aspect requires further exploration.

A heatmap visualization of the top twenty significantly altered metabolites was conducted (Figure 4b), complemented by mass spectrometry abundance charts for selected metabolites like glucose‐1‐P, galactinol, and D‐galactose‐6‐P, which demonstrated an upregulation in the spore‐exposed group (Figure 4e). Considering the established correlation between metabolites and lung microbiota, a provenance analysis of metabolites was performed on the MetOrigin website.^[^ ^51^ ^]^ The analysis revealed that among the detected metabolites, only a fraction was solely produced by the host, a significant number were co‐produced by the host and microbiota, while a considerable portion was exclusively generated by the microbiota (Figure 4c). This study also discovered substantial correlations between various microbes and metabolites within the galactose metabolism pathway (Figure 4d). On a genus level, metabolite D‐galactose‐6‐P showed significant positive correlations with *Staphylococcus* and *Lactobacillus*, and negative correlations with *Clostridium* and *Bacillus*. These findings provide crucial insights into the mechanisms by which fungal spore exposure influences polar metabolites in mouse lungs.

### 2.3 Impact of *F. Graminearum* on Macrophages in Fungal Pneumonia

**Figure 5:**
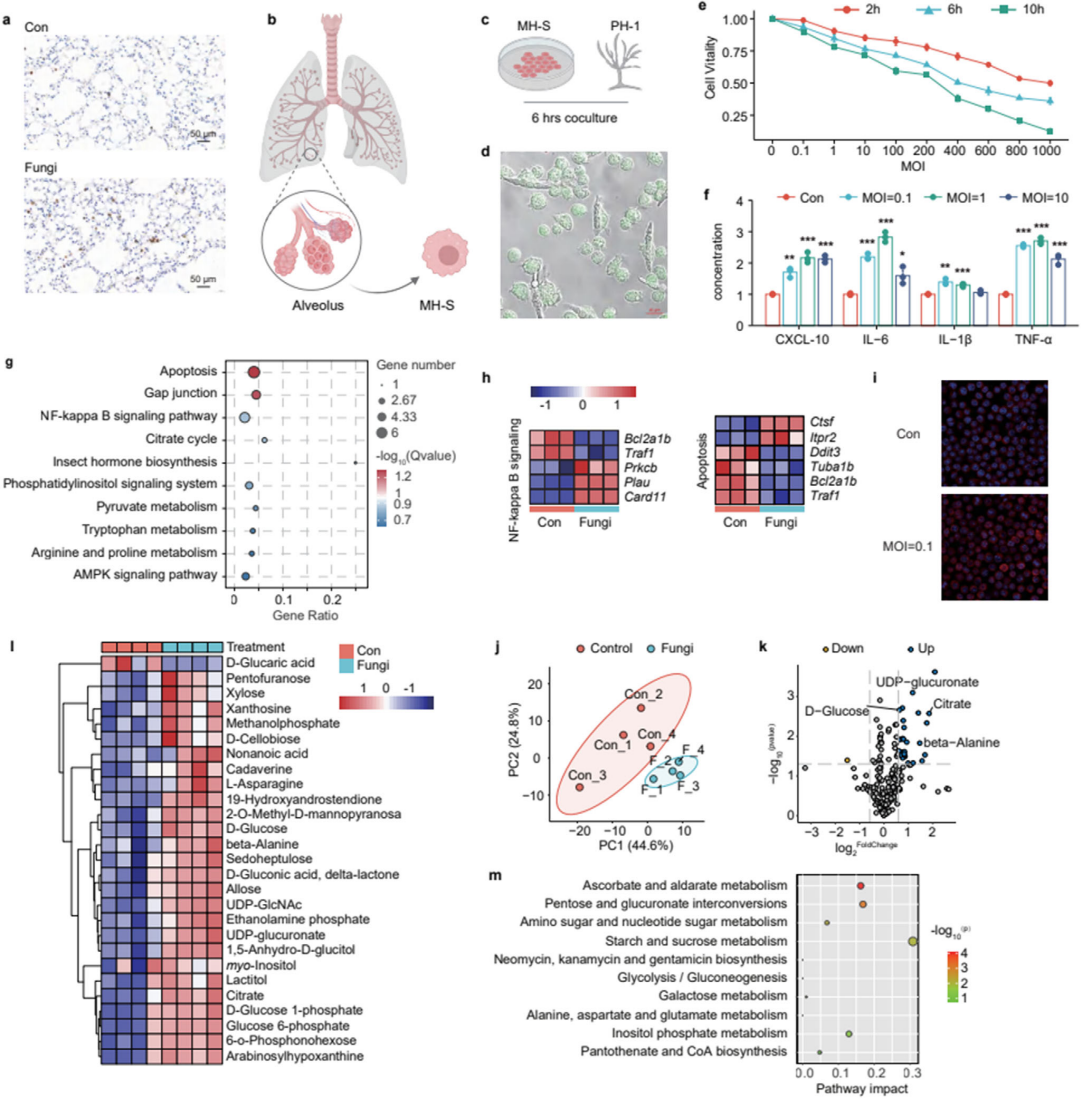
Transcriptome and metabolome alterations in mice alveolar macrophage MH‐S cells upon *F. graminearum* infection. a) immunohistochemical image of F4/80 in mouse lungs; b,c) schematic of MH‐S cell experiments; d) confocal microscopy photo of co‐cultured *F. graminearum* spores and MH‐S cells; e) MH‐S cell viability chart; f) expression of inflammation‐related mRNA in MH‐S cells (n = 3); g) transcriptomic pathway enrichment chart; h) gene expression chart for NF‐kappa B signaling and apoptosis pathways, with blue and red indicating downregulation and upregulation; i) confocal microscopy image of NF‐kB signaling P65 subunit nuclear translocation; j) PCA chart; k) volcano plot; l) heatmap of differential metabolites with red and blue indicating upregulation and downregulation; m) pathway enrichment chart of differential metabolites. Data are mean ± SEM. *, ** and *** denote *p*‐values < 0.05, < 0.01, and < 0.001, respectively.

In addition, our research utilized immunohistochemical analysis to mark F4/80, a macrophage marker. The analysis revealed an increase and aggregation of macrophages in the spore‐exposed group (**Figure** **5**a). This indicates that fungal pneumonia is accompanied by the occurrence of immune inflammation. To further validate these findings, we conducted in vitro experiments using the MH‐S murine alveolar macrophage cell line (Figure 5b). Initially, *F. graminearum* PH‐1 spores were co‐cultured with MH‐S cells for 6 h (Figure 5c), and subsequent observations under a confocal microscope, post‐calcein staining, revealed morphological alterations and aggregation of macrophages around the spores (Figure 5d). We then examined the impact of spores on MH‐S cell viability, noting a decrease in survival rate with increasing spore concentration and prolonged co‐culture duration (Figure 5e). Selecting a 6 h exposure period and varying spore concentrations (MOI = 0.1/1/10), RNA from the co‐cultured cells was extracted for RT‐qPCR analysis of inflammation‐related mRNA expression. This revealed a significant upregulation in the expression of the chemokine CXCL‐10 (*p* < 0.01), with other inflammatory markers also exhibiting varying degrees of elevation (Figure 5f). These results suggest that the co‐culture of PH‐1 spores with MH‐S cells triggered an inflammatory response in the macrophages, leading to their aggregation and subsequent inflammation. Further validation was sought through transcriptomic sequencing (Table S5, Supporting Information) and metabolomic identification (Table S6, Supporting Information) of MH‐S cells under a 6 h exposure to spores at a MOI of 0.1 (Figure 5c). Transcriptome sequencing highlighted gene transcription predominantly enriched in apoptosis and NF‐κB signaling pathways (Figure 5g), suggesting potential cell apoptosis due to spore infection. A heatmap of these pathways' differential genes is presented in Figure 5h.

To confirm the activation of the NF‐κB pathway, NF‐κB P65 subunit was marked, revealing its translocation from the cytoplasm to the nucleus in the spore‐infected group (Figure 5i; Figure S4, Supporting Information). These transcriptomic findings confirm the activation of the NF‐κB signaling pathway in MH‐S cells by spore infection, leading to induced apoptosis. After that, we performed metabolomics analysis of the co‐cultured cells (Figure S5, Supporting Information). The PCA showed significant metabolic alterations in MH‐S cells post spore infection (Figure 5j), with a volcano plot revealing overall metabolic changes (Figure 5k). The top 20 differentially expressed metabolites were visualized in a heatmap (Figure 5l), and input into the KEGG database for metabolic pathway analysis. The results indicated enrichment in multiple pathways related to galactose metabolism, including ascorbate and aldarate metabolism, pentose and glucuronate interconversions, starch and sucrose metabolism, glycolysis/gluconeogenesis, and amino sugar and nucleotide sugar metabolism (Figure 5m). These metabolomic outcomes provide further evidence for the influence of spore exposure on galactose metabolism pathways.

### 2.4 Homeostasis of Gut Microbiota on the Susceptibility to Fungal Pneumonia

In the preceding sections of our study, we conducted an exhaustive exploration of the role of pneumoniain metabolomic and microbiomic alterations, elucidating their association with the progression of respiratory diseases. While the lung microbiota plays a pivotal role in modulating local immune responses and metabolic equilibrium, the interplay of the gut‐lung axis is also of substantial significance. In the extended phase of our research, we delved into the impact of gut microbiota depletion on pneumonia and related metabolic pathways triggered by spore exposure.

**Figure 6:**
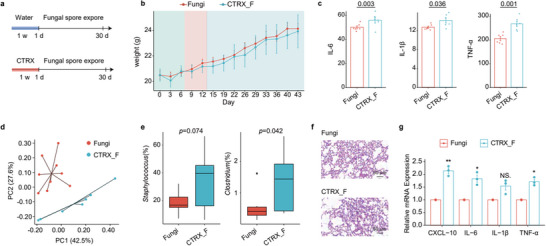
Susceptibility to fungal pneumonia and lung inflammatory indicators with gut microbiota imbalance in mice. a) experimental protocol diagram; b) body weight change graph, with green representing the adaptation period, red for antibiotic pretreatment, and blue for spore exposure period; c) changes in inflammatory cytokines IL‐6, IL‐1β, TNF‐α in BALF (n = 6); d) PCA of bacterial communities in BALF; e) Box plots of *Staphylococcus* and *Clostridium* abundance; f) H&E stained lung tissue; g) Expression of lung inflammation‐related mRNA. Data are mean ± SEM with * and ** indicating *p*‐values < 0.05 and < 0.01, respectively.

In our experimental setup, mice underwent a one‐week adaptation period followed by a seven‐day ceftriaxone sodium (CTRX) intervention to eradicate gut microbiota, with data supporting that CTRX alone did not alter pulmonary immune cell profiles,^[^ ^52^, ^53^ ^]^ subsequently leading to a thirty‐day spore exposure period (**Figure** **6**a; Figure S6, Supporting Information). Our findings revealed that mice in the CTRX_F group, having undergone antibiotic pre‐treatment, experienced a relative decline in body weight compared to the Fungi group (Figure 6b). Analysis of inflammatory cytokines IL‐6, IL‐1β, and TNF‐α in the BALF indicated a marked elevation in inflammation levels post‐antibiotic treatment in the CTRX_F group (*p* < 0.05, Figure 6c). The microbial composition in BALF also differed from that of the Fungi group (Figure 6d), with a notable increase in *Staphylococcus* and *Clostridium* (*p* < 0.05, Figure 6e). The H&E staining of lung tissues further corroborated these findings, revealing increased alveolar septal thickening, alveolar fusion, infiltration of inflammatory cells, and enhanced fibrosis (Figure 6f; Figure S7, Supporting Information). The results from the analysis of lung inflammation‐related mRNA were consistent with these observations (Figure 6g). From a phenotypic perspective, after the destabilization of the gut microbiota, the impact of *F. graminearum* on lung inflammation, pulmonary microbiota, and related negative effects became more pronounced.

**Figure 7:**
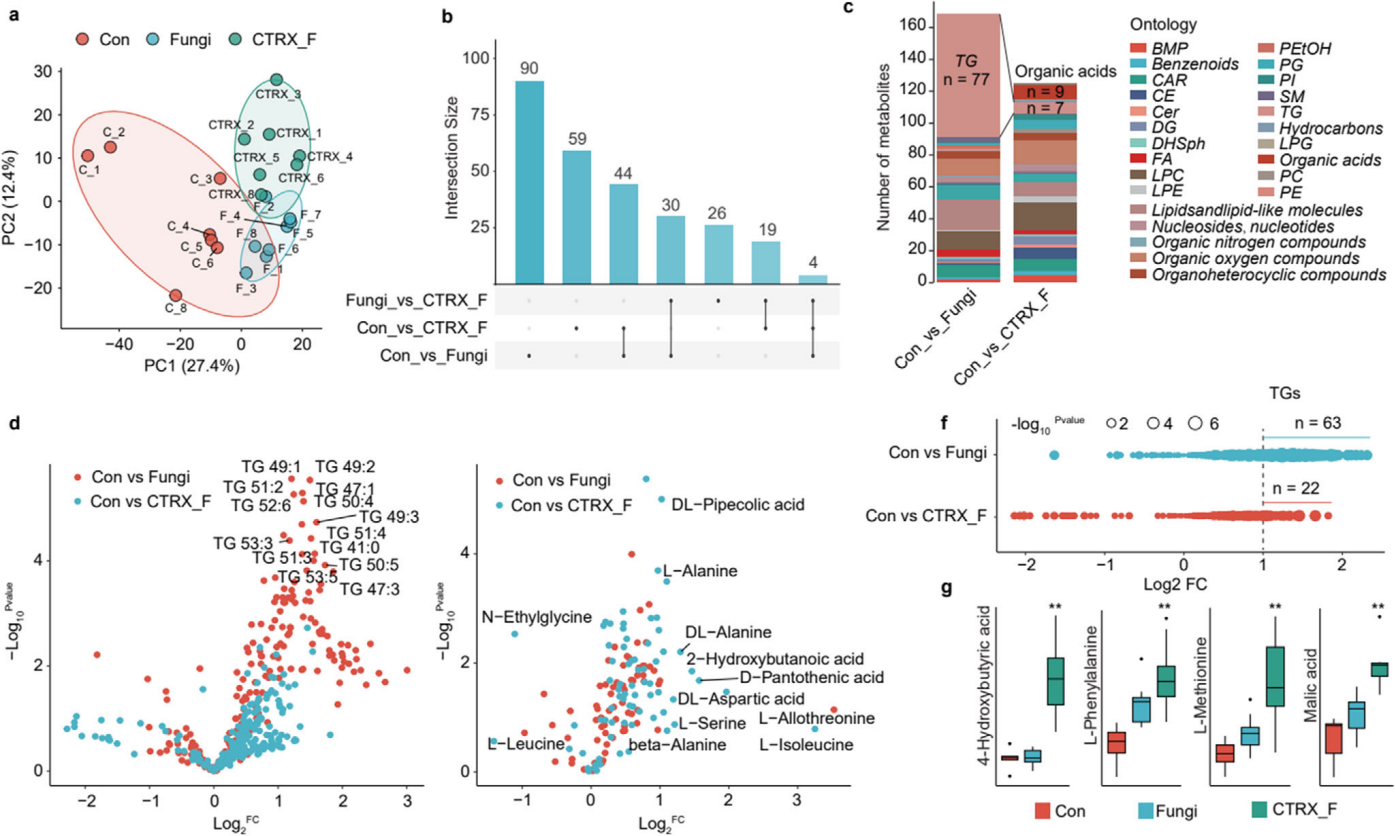
Lung metabolism in mice with fungal pneumonia subsequent to gut microbiota imbalance. a) PCA of metabolites; b) Intersection size graph showing common differential metabolites between three groups; c) Histogram displaying the quantity change of different types of differential metabolites; d,e) Volcano plots of differential metabolite changes; f) Bubble chart showing changes in differential TGs; g) Histogram showing changes in select organic acids. Data are mean ± SEM with * and ** denoting *p*‐values < 0.05 and < 0.01, respectively.

In our subsequent analysis, we scrutinized the lung metabolites (Table S7, Supporting Information). The PCA distinctly delineated the separation of metabolic characteristics among these groups (**Figure** **7**a). We employed the “Intersection Size” method to conduct statistical analysis on the differential metabolites between Con versus Fungi, Con versus CTRX_F, and Fungi versus CTRX_F, aiming to calculate the number of shared metabolites among them. We found that there were 44 more differential metabolites between the Con group and the Fungi group than between the Con group and the CTRX_F group. (Figure 7b). In terms of categories, the differential substances between the Antibiotic pre‐treated group and the Con group, as well as between the Fungi group and the Con group, were compared. It was found that the number of differential metabolites, particularly TGs, decreased significantly from a large amount to only 7, while the number of organic acids increased to 9 (Figure 7c). Volcano plots distinctly illustrated the specific changes in non‐polar metabolites (Figure 7d) and polar metabolites (Figure 7e) between the Fungi and CTRX_F groups. A detailed analysis of the significantly altered TGs revealed a decrease in upregulated differential TGs and an increase in downregulated ones following antibiotic intervention (Figure 7f). Among the polar metabolites, several organic acids and amino acids were found to be upregulated, including 4‐hydroxybutyric acid, L‐phenylalanine, L‐methionine, malic acid, and others (Figure 7g). These results underscore the significant impact of gut microbiota depletion on pneumonia and metabolic pathways induced by spore exposure.

### 2.5 Blood Mediates Gut Microbiota in regulating Fungal Pneumonia Susceptibility

**Figure 8:**
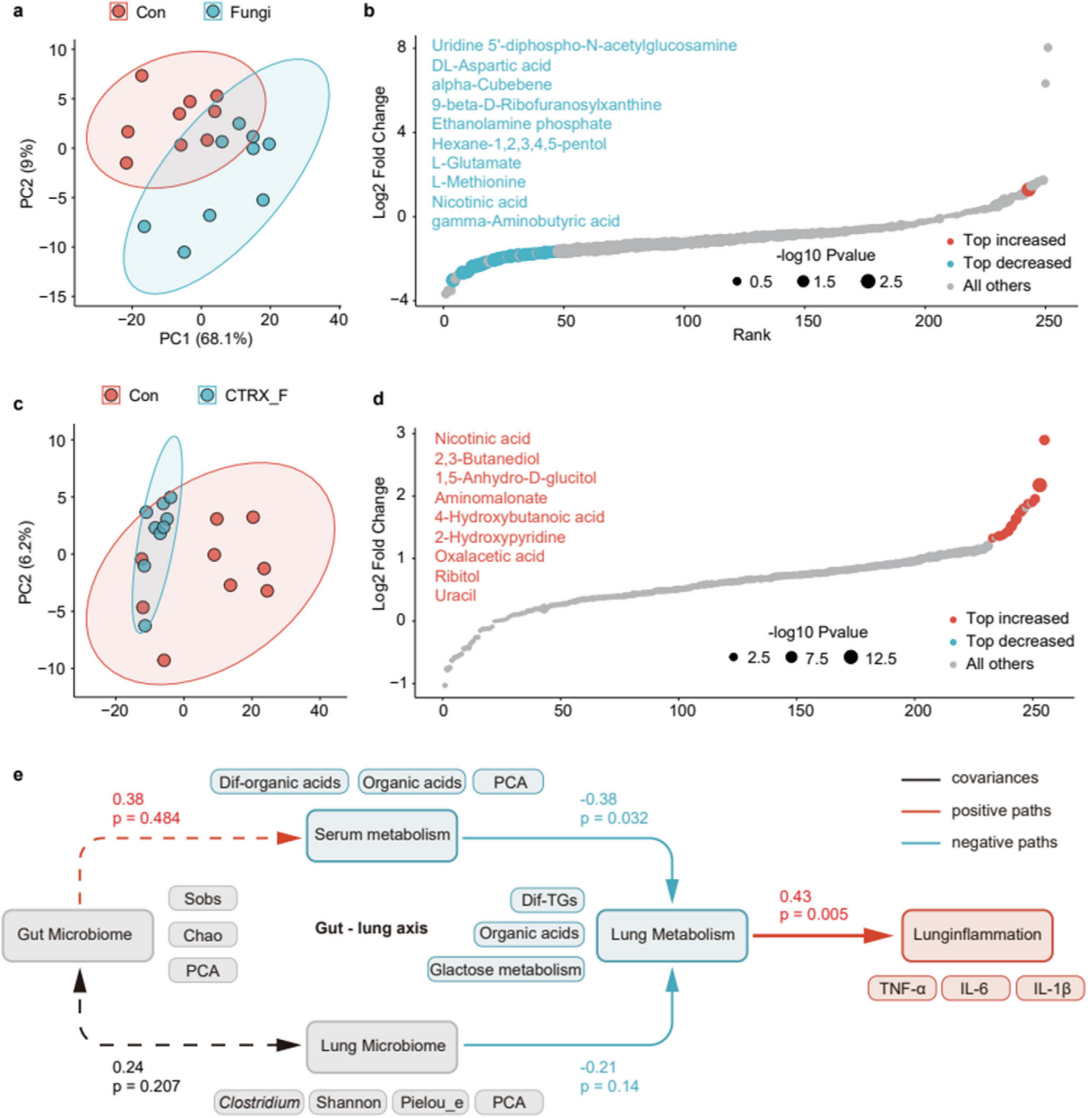
Blood serves as an intermediary to mediate the susceptibility of fungal pneumonia affected by the gut microbiota. a) PCA of serum metabolites comparing the Con and Fungi groups; b) Scatter plot of serum metabolites comparing the Con and Fungi groups; c) PCA of serum metabolites comparing the Con and CTRX_F groups. d) scatter plot of serum metabolites comparing the Con and CTRX_F groups; e) Structural equation model diagram.

As the above conclusion supports the notion that changes in gut microbiota can influence the metabolism and inflammation of *F. graminearum* in the mice lungs, we began to explore the complex interactions between gut microbiota, lung inflammation, metabolomics, and microbiology, with a particular emphasis on the mediating role of blood in this connection. By delving into the blood metabolome (Table S8, Supporting Information), our aim was to gain deeper insights into the nuances of the gut‐lung axis. Experimental findings indicated significant differences in blood metabolic characteristics between the spore‐exposed mice (Fungi group) and the control group (Con group), as depicted in **Figure** **8**a. In contrast, the differences between the antibiotic‐pre‐treated CTRX_F group and the Con group were relatively minor (Figure 8c). Differential analysis of all metabolites in the blood revealed a substantial downregulation in the Fungi group (Figure 8b), while a notable upregulation was observed post‐antibiotic treatment (Figure 8d), predominantly involving organic acid metabolites. We hypothesized that pneumonia is influenced by lung‐specific metabolic characteristics, which in turn are affected by both blood metabolites and lung microbiota, with blood metabolism itself being modulated by gut microbiota. To test this hypothesis, we employed structural equation modeling (SEM) for analysis (Figure 8e and Table S9, Supporting Information). In the model, path coefficients ≈0.5 indicated high effects, ≈0.3 medium effects, and ≈0.1 low effects.

We selected several measurable manifest variables to represent five latent variables that are challenging to directly measure: lung inflammation, lung metabolism, lung microbiota, blood metabolism, and gut microbiota. Specifically, TNF‐α, IL‐6, and IL‐1β levels in the BALF were used to represent lung inflammation; enrichment of the galactose metabolism pathway's z‐score, differential triglycerides content (Dif‐TGs), and organic acid levels were employed to signify lung metabolism; Clostridium's OTU values, Shannon index, Pielou's e index, and PCA's PC1 were chosen to characterize lung microbiota; organic acid levels, differential organic acid content (dif‐organic acid), and PCA's PC1 were used for blood metabolism; and the Sobs index, Chao index, and PCA's PC3 were applied to represent gut microbiota. The analysis outcomes revealed a path coefficient of 0.43 between pneumonia and lung metabolism, −0.21 between lung metabolism and lung microbiota, −0.38 between lung metabolism and blood metabolism, and 0.38 between blood metabolism and gut microbiota. Additionally, a covariance of 0.24 was noted between gut and lung microbiota. These findings unveil the complex interactions within the gut‐lung axis in regulating pneumonia and metabolism, highlighting the critical mediating role of blood metabolism in bridging the gut and lung microenvironments.

## 3 Discussion

Spore contamination is serious and widespread, affecting food security and public health, and current studies have focused on the pulmonary toxicity, hepatotoxicity, damage and inactivation of fungal spores in healthy populations.^[^ ^54^, ^55^, ^56^ ^]^ However, lung inflammation caused by long‐term exposure of immunocompromised individuals and healthy populations to this agricultural environment should not be ignored, so in this paper, we constructed two different exposure models (immunosuppression exposure model and subacute exposure model) to analyze in depth the potential health threat of *F. graminearum* PH‐1 spores exposure through the nasal‐lung pathway, which provides an important insights for health risk assessment, prevention and control in the food processing environment. In our study, we employed an intratracheal injection of *F. graminearum* PH‐1 spores to establish a model for fungal spore exposure via the sinus‐lung route. Utilizing PASM staining, we were able to observe the colonization of *F. graminearum* in the lungs. Body weight, as a sensitive indicator of animal health status,^[^ ^57^ ^]^ was employed to assess the potential toxic effects of exogenous substances on organisms. Notably, we observed a significant decrease in the body weight of mice after 30 days of spore exposure. Additionally, through H&E staining of the lungs, our objective was to explore whether the invasion of *F. graminearum* could induce inflammation and fibrosis. The experimental results indicated that spore exposure exacerbated lung inflammatory responses, potentially signaling the early onset of certain lung diseases. Analysis of BALF ELISA results and relative mRNA expression levels in lung tissues revealed a significant increase in lung concentrations of IL‐6, TNF‐α, and IL‐1β upon spore exposure, alongside the activation of chemotactic cytokines such as CXCL‐10, which facilitate the recruitment of immune cells like alveolar macrophages and the activation of immune responses. Cell experiments further confirmed that spore exposure activated multiple inflammation‐related pathways in alveolar macrophages, including the key inflammatory regulatory pathway – NF‐*κ*B,^[^ ^58^ ^]^ and the caspase cascade pathway leading to cell apoptosis triggered by TNF‐α.^[^ ^59^ ^]^ Persistent pneumonia may lead to various lung diseases, including lung fibrosis,^[^ ^60^ ^]^ asthma,^[^ ^61^ ^]^ chronic obstructive lung disease (COPD), and bronchiectasis.^[^ ^62^ ^]^

Our study results revealed that under the influence of fungal spore exposure, there was a significant reduction in the diversity and evenness of lung microbial communities, leading to the formation of a microbiota distinct from its original state. Notably, we observed an induced enrichment of specific lung bacteria, particularly a marked increase in the genus *Staphylococcus*, which has been previously identified as a potential contributor to secondary pneumonia in altered lung microenvironments following influenza infection.^[^ ^63^ ^]^ Furthermore, the activation of the NF‐*κ*B pathway, a primary inflammatory response route, has been associated with pneumonia and damage caused by Methicillin‐resistant *Staphylococcus aureus*.^[^ ^64^ ^]^ Concurrently, the significant reduction of the *Clostridium* genus in our study raises concerns, as it is considered a beneficial bacterium playing a crucial role in resisting influenza virus pneumonia,^[^ ^35^ ^]^ lung cancer immunotherapy,^[^ ^36^, ^37^ ^]^ and neurological regeneration.^[^ ^38^ ^]^ Its reduction could potentially disrupt lung immune responses, exacerbating lung inflammation. These findings underscore the significance of lung microbial composition in maintaining health and responding to diseases, especially under external stresses like fungal spore exposure.

Furthermore, we observed significant changes in lung metabolism following fungal pneumonia. Through metabolomics analysis of mice lung tissues, we identified 920 non‐polar metabolites, predominantly lipids, and 225 polar metabolites. Non‐polar metabolites are intimately linked to inflammatory processes, especially in modulating macrophage production of IL‐1β, which involves the influence of histone deacetylase 3 on lipid metabolism, particularly its limitation on exogenous fatty acid‐supported fatty acid oxidation.^[^ ^65^ ^]^ Post spore exposure, we detected a significant increase in lung TGs, which might be attributed to macrophages accumulating TGs in intracellular lipid droplets in response to inflammation. This accumulation could result from a reduction in Adipose Triglyceride Lipase (ATGL) protein levels due to hypoxia‐inducible lipid droplet‐associated protein upregulation, thereby decreasing ATGL‐mediated triglyceride hydrolysis.^[^ ^66^ ^]^ Additionally, the Triglyceride‐Glucose index has been linked to respiratory symptoms, chronic bronchitis, and restrictive lung function impairment.^[^ ^67^ ^]^

In terms of polar metabolites, our study observed that fungal spore exposure significantly influenced lung sugar metabolism pathways, particularly galactose metabolism and the pentose phosphate pathway. Under normal physiological conditions, D‐galactose (D‐Gal), as a reducing sugar, is efficiently converted into glucose to meet the body's energy requirements.^[^ ^68^ ^]^ However, excessive accumulation of D‐Gal can induce oxidative stress, leading to oxidative damage of cellular lipids, proteins, and nucleic acids, thereby impacting cellular functions and provoking cellular injury.^[^ ^69^ ^]^ The oxidation of D‐Gal generates free radicals that not only damage cellular structures but also react with the free amino groups of proteins and peptides, forming advanced glycation end products (AGEs).^[^ ^70^ ^]^ These AGEs are highly associated with COPD and the decline of lung function,^[^ ^71^ ^]^ and may exacerbate lung complications related to diabetes.^[^ ^72^ ^]^ Excess monosaccharides can promote ectopic lipid accumulation, simultaneously activating immune cells, leading to low‐grade inflammation.^[^ ^73^, ^74^, ^75^ ^]^ Therefore, the changes in sugar metabolism pathways, especially the alteration of the galactose metabolism pathway induced by fungal spore exposure, may lead to a range of inflammatory injuries in the lungs.

The results of our study underscore the close association between the lung metabolome and microbiome following fungal spore exposure. We found that these changes are not solely induced by the *F. graminearum*, but rather are the result of interactions between the host and microbial communities. Through retrospective analysis, we revealed the close connections among these disturbances, suggesting that they may occur through interactions, rather than independently of the *F. graminearum*. Our exploration into the causal relationships between these changes, particularly focused on whether microbial dysbiosis leads to metabolic disorders, or whether lung injury caused by fungal spore exposure alters metabolic disorders, thus leading to microbial dysbiosis.^[^ ^76^ ^]^ Previous studies have indicated that disruption of the microbial community can lead to chronic lung inflammation, which in turn may cause changes in microbial abundance, exacerbating the imbalance between microbiota and metabolites, forming a positive feedback loop.^[^ ^76^, ^77^, ^78^ ^]^ These findings highlight the importance of considering the interplay between microbial communities and host metabolism in disease research, offering new insights into the treatment and prevention of lung diseases caused by fungal spore exposure.

A salient discovery in our study is the observed amelioration of a range of lung injury markers induced by *F. graminearum* following the eradication of gut microbiota with antibiotics. Under the regimen of antibiotic pre‐treatment, a substantial reduction in the number of lung TGs was noted, decreasing sharply, while there was an increase in the number of organic acids. These findings lend support to the concept of the lung‐gut axis, a notion corroborated by multiple studies.^[^ ^79^, ^80^, ^81^ ^]^ Research has shown that pro‐inflammatory mediators in the lungs, such as cigarette smoke, may partially migrate to the intestines via the bloodstream, exacerbating intestinal damage and inducing inflammation, epithelial barrier disruption, and oxidative stress. Additionally, it has been suggested that pro‐inflammatory mediators produced by lung cells (e.g., TNF‐α, IFN‐γ, IL‐6, IL‐8, CRP, and ROS) may activate innate immune responses, thereby impacting intestinal health. Conversely, damage to intestinal functions can not only increase the production and circulation of pro‐inflammatory mediators but also weaken nutrient absorption, antioxidative capabilities, and defenses against pathogens and other environmental stimuli. In our study, alterations in the gut microbiota appear to precipitate changes in lung inflammation, microbiome, and metabolome, potentially by influencing blood metabolism and consequently lung metabolism. As illustrated in **Figure** **8**, changes in gut microbiota affect blood metabolism, which in turn influences lung metabolism. Changes in the lung microbiome might also impact lung metabolism, ultimately leading to lung inflammation. Moreover, previous research has also proposed a potential connection between lung and gut microbiota,^[^ ^80^ ^]^ providing additional evidence supporting our findings and further underscoring the significance of the lung‐gut axis in lung health and disease. Fortunately, due to the strong self‐cleaning ability of the lungs, we found that lung metabolism (Table S10, Supporting Information) and serum metabolism recovered in the normal feeding process for the following 30 days of the experiment, which was reflected in the recovery of TG (Figure S7, Supporting Information). This may be related to the recovery of the gut microbiota.

Future studies should consider using germ‐free or symbiont depleted mice to study fungal pneumonia to further determine the relationship between the lung microbiome and metabolic profile in mice, as well as whether the role of the lung microbiome on lung injury is individual, additive, or antagonistic. Antibiotics of different categories or with different mechanisms of action have different effects on the structure of the gut microbiota, and their effects on pneumonia are also different, which can be used as a follow‐up study. The addition of a nonpathogenic *F. graminearum* mutant group would have helped determine whether the observed pulmonary inflammation, microbiota changes, and metabolic alterations were due to the fungus's pathogenicity or other aspects of exposure.

Current clinical interventions targeting respiratory health based on the gut‐lung axis theory primarily employ dietary modifications and probiotic supplementation strategies.^[^ ^82^ ^]^ Our study revealed that fungal spore exposure leads to a significant decrease in pulmonary *Clostridium* abundance. Given that *Clostridium*, as a dominant gut microbiota, exerts multiple physiological functions through metabolite secretion and immunomodulation pathways,^[^ ^83^ ^]^ our subsequent research will specifically investigate the therapeutic effects of *Clostridium* supplementation on alleviating fungal pneumonia symptoms. Furthermore, we will explore mitigating fungal spore‐induced pulmonary pathological damage by modulating the NF‐κB signaling pathway. However, long‐term application of gut microbiota modulation therapies may raise several clinical safety concerns, including secondary dysbiosis and increased risk of opportunistic infections. Additionally, the interaction mechanisms between gut microbiota modulators and conventional antifungal drugs, along with their clinical implications, represent another critical area for investigation. We will systematically analyze their potential pharmacodynamic interactions and propose personalized medication recommendations.

Our studies prompt the public to pay attention to the balance of the gut microbiota since it is crucial for preventing pneumonia, especially fungal pneumonia. Researchers and medical practitioners should also concentrate on the relationship between microbiota and immunity, and explore new therapeutic methods such as modulating the gut microbiota for the prevention and treatment of pneumonia.

## 4 Conclusions

In summary, our research demonstrates the significant role of gut microbiota in fungal pneumonia. The gut microbiota is involved in modulating susceptibility to fungal pneumonia through the gut‐lung axis, influencing lung metabolism and inflammatory responses. Antibiotic‐induced disruption of the gut microbiota leads to enhanced colonization of *F. graminearum* in the lungs and exacerbated lung injury. The observed changes in lung and gut microbiota and associated metabolism highlight the complex interplay between these systems. Importantly, our findings emphasize the need for public awareness regarding the importance of gut microbiota balance in preventing fungal pneumonia and encourage further research and exploration of microbiota modulation as a potential therapeutic strategy for pneumonia prevention and treatment. Overall, this work contributes to a deeper understanding of the intricate relationship between microbiota and fungal pneumonia and paves the way for future investigations and interventions in this field.

## 5 Experimental Section

### Fungi

In this study, the standard *F. graminearum* PH‐1 strain was utilized. Under a continuous light intensity of 1000 µmol m^2^·s, the *F. graminearum* PH‐1 strain was inoculated onto potato dextrose agar (PDA) medium and subsequently incubated in a 26 °C incubator for 7 days. Following the incubation period, the colony was repeatedly washed and disrupted using 5 mL of phosphate‐buffered saline (PBS) containing 0.05% Tween‐80. The fungal suspension was then filtered through three layers of sterile gauze to obtain a fungal spore suspension. Finally, quantitative analysis of the spore suspension was performed using a hemocytometer with a 10‐fold dilution factor.

### Mice

Fifty male BALB/c nude mice, weighing 18–22 g and aged 6 weeks, were acquired from Hangzhou Ziyuan Experimental Animal Technology Co., Ltd. After a 7‐day acclimatization period at the facilities of Shanghai Baiyou Biotechnology Co., Ltd., the mice were maintained under strict environmental control, including a 12 h light/dark cycle, for all experimental procedures.

The mice underwent a 7‐day adaptation period before being randomly divided into three groups: the control (Con) group, the fungal spore (Fungi) group, and the ceftriaxone (CTRX_F) group. In brief, the Con group received normal feed and tracheal injections of PBS (20 µL, 0.01 M), while the Fungi group was injected weekly with *F. graminearum* spore suspensions. The CTRX_F group received twice‐daily oral administration of CTRX (200 µL, 400 mg mL^−1^) from the first to the seventh day post‐adaptation in order to induce gut microbiota depletion. On day 14, the fungal spore and CTRX_F groups were injected with a 2 × 10^5^ spores mL^−1^ suspension (20 µL), with injections continuing weekly for thirty days. On day 43, ten mice from each group were studied for changes in various parameters after thirty days of fungal spore intake, labeled as, Con group, Fungi group, and CTRX_F group.

### Sample Collection

Biological samples were systematically collected at the time of euthanasia for all mice. Blood was obtained via orbital venous plexus puncture. Samples designated for fixation were immediately immersed in 4% paraformaldehyde solution. Bronchoalveolar lavage fluid (BALF) was collected by ligating the left lung and lavaging the right lung thrice with sterile PBS. The collected BALF was then centrifuged at 11 000 rpm for 15 min at 4 °C.

### Cell Viability and CLSM

Following infection with varying concentrations of *F. graminearum* PH‐1 spores at different time points, live cells were labeled using Calcein Acetoxymethyl Ester (Calcein AM). The fluorescence intensity was measured using a BioTek H1 type microplate reader, with an excitation wavelength of 490 nm and emission wavelength of 520 nm. Additionally, for a specific time point of 6 h and a multiplicity of infection (MOI) of 0.1, representing a 10:1 ratio of cells to spores, cells were stained with Calcein AM and imaged using a LSM880 confocal microscope (Carl Zeiss, Germany). The P65 subunit was subjected to cy3 staining using Biyun Tian's NF‐κB Activation and Nuclear Translocation Assay Kit in order to assess the activation of NF‐κB nuclear transport. Subsequently, they were imaged using a Lycra confocal microscope.

### qPCR

Following infection with various concentrations of *F. graminearum* PH‐1 spores at different time intervals, MH‐S cells were processed for RNA extraction using the Total RNA Isolation Kit from Novozan Biotech, Nanjing. The extracted RNA was reverse‐transcribed to cDNA with HiScript III RT SuperMix for qPCR (Novozan Biotech). mRNA expression levels were quantified using ChamQ Universal SYBR qPCR Master Mix (Novozan Biotech). Primer sequences were as follows: Tubulin, F: 5′‐GAAGCAGCAACCATGCGTGA‐3′, R: 5′‐ CCTCCCCCAATGGTCTTGTC‐3′. CXCL‐10, F: 5′‐CAACTGCATCCATATCGATGAC, R: 5′‐GATTCCGGATTCAGACATCTCT‐3′. IL‐6, F: 5′– GACAAAGCCAGAGTCCTTCAGA‐3′, R: 5′‐ TGTGACTCCAGCTTATCTCTTGG‐3′. IL‐1β, F: 5′‐ TGCCACCTTTTGACAGTGATG‐3′, R: 5′‐ TGATGTGCTGCTGCGAGATT‐3′. TNF‐α, F: 5′‐ CCTGTAGCCCACGTCGTAG‐3′, R: 5′‐ GGGAGTAGACAAGGTACAACCC.

### ELISA

The right lung was lavaged thrice with sterile PBS, and the lavage fluid was collected. The levels of inflammatory cytokines in the BALF were measured using an ELISA kit for IL‐6, IL‐1β, and TNF‐α, provided by Nanjing Senberga Biotechnology Co., LTD.

### r RNA Sequencing

Adopting the protocol established by Chen et al. (2018), it was extracted genomic DNA from mouse BALF using the Genomic DNA Mini Preparation Kit, as provided by Beyotime Institute of Biotechnology, Shanghai, China. The PCR products were sequenced on a single lane of the Illumina MiSeq platform at Personal Biotechnology Co., Ltd., Shanghai, China.

### Metabolites Extraction and Detection

In this metabolomic analysis of mouse lung tissues and serum, we employed a streamlined and non‐selective extraction approach for optimal coverage and throughput. Lung tissue (50 mg ± 5 mg) was processed in a 2 mL Eppendorf tube with 225 µL of methanol (MeOH), 750 µL of methyl tert‐butyl ether (MTBE), and 2 mm zirconium beads. This mixture was vortexed for 10 s, homogenized using a high‐throughput tissue grinder, sonicated for 5 min in an ice‐water bath, and then mixed with 188 µL of LC/MS‐grade water. After vortexing for 10 s again, the sample was centrifuged at 12 300 rpm for 5 min at 4 °C. Subsequently, 350 µL aliquots of the upper non‐polar phase and 250 µL aliquots of the bottom polar phase were collected and placed in a vacuum freeze‐concentrating dryer for 8 h until the samples were completely evaporated. Remaining fractions were combined to form QC pools and were injected after every set of 10 biological samples.

The upper liquid extracted with MTBE as described above was evaporated to complete dryness, and the dried samples were resuspended in 110 µL of reconstituted stock solution (methanol: toluene = 9: 1), centrifugation, bottling and for LCMS detection. The bottom polar phase was then derivatized by dissolving the sample in 80 µL of 20 mg mL^−1^ methoxyamine hydrochloride (MeOX) solution, vortex‐mixed for 30 s, and placed in a constant temperature drying oven at 60 °C for 60 min. Add 100 µL of Bis (trimethylsilyl) trifluoroacetamide (BSTFA) solvent to each sample (including QC) in a fume hood, vortex for 30 s, and then put it into a constant temperature drying oven at 70 °C for 90 min. Add 5 µL of fatty acid methyl esters (FAMEs) to each QC sample. After the samples were cooled to room temperature, centrifuge at 12 000 r min^−1^ at 4 °C for 10 min. Pipette the supernatant into a glass bottle and store at 4 °C for GC‐TOF/MS detection.

Serum samples (50 µL) were extracted using a 1 ml mixture of acetonitrile, isopropanol, and water (3:3:2, v:v:v). This was followed by a 10 min sonication in an ice‐water bath, 10 s vortexing, and centrifugation at 12 300 rpm at 4 °C for 10 min. The 400 µL supernatant was then lyophilized and underwent identical derivatization as the lung tissue samples. Throughout these procedures, lung tissues and serum were consistently maintained on ice.

### Nontarget LCMS Metabolomics Analysis

For lipidomics analysis, 3 µL of the resuspended non‐polar phase was injected into a Vanquish UHPLC system (Thermo Scientific, Waltham, MA, USA) equipped with a Waters Acquity UPLC HSS T3 column (1.8 µm, 2.1 × 100 mm) coupled with a Waters Acquity VanGuard CSH C18 precolumn (5 mm × 2.1 mm id; 1.7 µm). The oven temperature and flow rate were set at 65 °C and 0.6 mL min^−1^, respectively. The Thermo Scientific Q Exactive Focus benchtop LC‐MS/MS combines quadrupole precursor ion selection and a high‐resolution accurate‐mass (HR/AM) Orbitrap mass analyzer was used to collect spectra with a data‐dependent MS/MS spectra acquisition method. The ion source parameters were configured as follows: spray voltage was set at 3.6 kV; sheath gas flow rate was adjusted to 60 arbitrary units; auxiliary gas flow rate was designated as 25 arbitrary units; sweep gas flow rate was established at 2 arbitrary units; capillary temperature was maintained at 300 °C; S‐lens RF level was set to 50; and auxiliary gas heater temperature was regulated to 370 °C. The following acquisition parameters were used for MS1 analysis: resolution, 60 000, AGC target, 1e6; Maximum IT, 100 ms; scan range 150–1700 m/z; spectrum data type, centroid. Data dependent MS/MS parameters: resolution, 15 000; AGC target, 1e5; maximum IT, 50 ms; loop count, 3; TopN, 3; isolation window, 1.0 m/z; fixed first mass, 70.0 m/z; (N)CE/ stepped nce, 20, 30, 40; spectrum data type, centroid; minimum AGC target, 8e3; intensity threshold, 1.6e5; exclude isotopes, on; dynamic exclusion, 3.0 s.

### Nontarget GCMS Metabolomics Analysis

Cellular metabolites were detected using the GC/MS‐TOF coupled instrument. A DB‐5 MS column (30 m × 0.25 mm × 0.25 µm, Agilent, USA) was utilized. The chromatographic conditions were as follows: injection volume of 1 µL, helium as the carrier gas, initial temperature at 70 °C, retention time of 1 min, followed by ramping to 280 °C at a rate of 6 °C min^−1^, and a 5 min hold. The mass spectrometry analysis employed the following conditions: negative ion mode, 1 mA emission current, 300 °C interface temperature, 230 °C emission source temperature, 2000 V detection voltage, scan rate of 10 spec / s, electron energy of – 70 eV, and a solvent delay of 3 min.

### RNA Sequencing

Total RNA was isolated from the MH‐S by TRIzol reagent. The paired‐end library was synthesized using the TruSeq RNA Sample Preparation Kit (Illumina, SanDiego, USA) according to the preparation guide. The libraries were sequenced on the Illumina Hiseq X‐ten platform. The extended protocol detail sand procedures of the data analysis were all provided by GENE DENOVO (Guangzhou, China).

### H&E Staining and PASM Staining

Haematoxylin and Eosin staining (H&E staining): Sections are dewaxed in eco‐friendly dewaxing solutions I and II for 20 min each, followed by dehydration through a series of alcohol (5 min each in absolute ethanol I and II, then 5 min in 75% alcohol), and washed with tap water. Hematoxylin staining involves immersing sections in hematoxylin for 3–5 min, rinsing with tap water, differentiating, returning blue, and rinsing under running water. Eosin staining was preceded by dehydration in 85% and 95% alcohol for 5 min each, followed by eosin staining for 5 min. The sections were then dehydrated in a series of absolute ethanol (5 min 75% alcohol, then 5 min each in absolute ethanol I and II), cleared in xylene I and II for 5 min each, and mounted in neutral resin.

For the Periodic Acid – Schiff with Methenamine Silver staining (PASM staining), this methodology entailed a meticulous process beginning with the dewaxing of paraffin‐embedded sections. This was achieved through sequential immersion in xylene (20 min each in xylene I and II), followed by rehydration in a graded ethanol series (5 min each in absolute ethanol I and II, succeeded by 5 min in 75% alcohol). Subsequently, the sections were thoroughly rinsed with both tap and distilled water. The sections were then immersed overnight in PASM solution A and briefly rinsed with water. Tissue acidification was accomplished using PASM solution B for 15 – 20 min, followed by 3–5 washes with distilled water. The PASM working solution, essential for the staining process, was prepared by mixing PASM solutions C (in a 20:1 ratio) and E with distilled water. Sections were incubated with this preheated mixture at a temperature range of 56 – 59 °C. Following 40 min of incubation, microscopic observations were performed at 10 min intervals, with the appearance of black‐stained fungal structures in lung tissue serving as the endpoint criterion. This was followed by three washes with distilled water. Optionally, sections could be briefly treated with PASM solution F and counterstained for 20 s in PASM solution F after dehydration with 85% and 95% alcohol. The final step involved dehydration through a series of ethanol and xylene, culminating in mounting in neutral resin. All reagents for this procedure were provided by Shanghai Biyuntian Biotechnology Co., Ltd. The overall pathological changes of the three groups were shown in the attached Figure S3 (Supporting Information).

### Date Processing

All the LC‐MS raw data files were converted into ABF format using ABF converter (https://www.reifycs.com/AbfConverter/). MS‐DIAL ver.4.00 software was used for peak picking, annotation and alignment. The detailed parameter setting was as follows: MS1 tolerance, 0.005 Da; MS2 tolerance, 0.01 Da; minimum peak height, 20 000 amplitude; mass slice width, 0.1 Da; smoothing method, linear weighted moving average; smoothing level, 5 scans; minimum peak width, 10 scans. [M+H]^+^, [M+NH_4_]^+^, [M+Na]^+^, [2M+H]^+^, [2M+NH_4_]^+^, and [2M+Na]^+^ were included in adduct ion setting for positive mode lipidomics analysis, [M‐H]^−^, [M+Cl]^−^, and [M+Hac‐H]^−^ for negative mode lipidomics. Compounds were annotated by accurate precursor masses and MS/MS spectra against libraries in LipidBlast.

All the GCTOF raw data were conveted to.cdf format through LECO chromTOF, the coverted to ABF format using ABF converter. MS‐DIAL ver.4.00 software was used for peak picking, deconvolution, annotation, and alignment. The detailed parameter setting was as follows: MS1 tolerance, 0.5 Da; minimum peak height, 5000 amplitude; smoothing method, linear weighted moving average; smoothing level, 5 scans; minimum peak width, 30 scans. Compounds were annotated EI spectra against Fiehnlib, with tolerance 0.7, and retention index (FAMEs) tolerance 8000.

### Statistical Analysis

Statistical analysis was performed by log transformation, and Pareto scaling. PCA was used for multivariate statistics and visualization, specifically for outlier detection. All results are shown as the mean ± SEM. ANOVA and Duncan's test for multiple comparisons were used to analyze data. The visualization of metabolites is performed using the R package, ggplot, ggpubr, pheatmap, and ropls. Microbiological analyses were performed using the statistical software R. Spearman rank correlation analyses and fold change calculations were conducted using R. Structural equation modeling analysis was performed software R (mediation), based on nonsignificant chisquare test. Structural equation modeling (SEM) was carried out using the software AMOS. Before running the model, the data of lung inflammation, lung metabolism, lung microbiome, serum metabolism, and gut microbiome were subjected to Z‐score processing.

## Conflict of Interest

The authors declare no conflict of interest.

## Author Contributions

J.J., X.S. designed the research. J.J. carried out the analysis. W.B. performed the experimental studies. W.B., Y.W. performed analysis of microbiota. X.S supervised the work. J.L. performed analysis for lung metabolism. J.J. and B.W. wrote the paper with contributions from all other authors. J.P., Y.Z., J.Y., Y.Y., L.S. and J.D., contributed to the statistics and grammar.

## Supporting information



Supporting Information

Supplemental Table 1

Supplemental Table 2

Supplemental Table 3

Supplemental Table 4

Supplemental Table 5

Supplemental Table 6

Supplemental Table 7

Supplemental Table 8

Supplemental Table 9

Supplemental Table 10

## Data Availability

The data that support the findings of this study are available from the corresponding author upon reasonable request.
