# Supplementary material for: Homeostasis of Gut Microbiota Protects against Susceptibility to Fungal Pneumonia
Source: Adv Sci (Weinh). 2025 Aug 4;12(40):e16455. doi: 10.1002/advs.202416455 (PMC12561360; doi:10.1002/advs.202416455)
Supplement: Supplementary file 1 — Supporting Information [file ADVS-12-e16455-s010.docx]

Supporting Information

**Homeostasis of Gut Microbiota Protects Against Susceptibility to Fungal Pneumonia**

Jian Ji 1, 2, 8, Yongli Ye 1, 2, 8, Jiadi Sun 1, 2, 8, Lina Sheng 1, 2, 8, Jinyou Li 3, Jin Yang 3, Bing Wu 1, 2, 8, Yuting Wang 1, 2, 8, Xingxing Gao 1, 2, 8, Liang Luo 4, Jianfeng Ping 5, 6, Yinzhi Zhang 7, and Xiulan Sun 1, 2, 8*

**Figures List：**

**Figure S1.** Lung tissue images of infected and normal mice revealing hemorrhagic areas caused by F. graminearum infection.

**Figure S2.** **Changes of polar and non-polar metabolites in lung.** **A** Nonpolar metabolite species distribution map; **B** Polar metabolite species change distribution map.

**Figure S3** **The number of carbon atoms and unsaturated bonds of CAR, LPC, TG and SM.** **A** The number of carbon atoms and the number of unsaturated bonds of CAR. **B** The number of carbon atoms and the number of unsaturated bonds of LPC. **C** The number of carbon atoms and the number of unsaturated bonds of TG. **D** The number of carbon atoms and the number of unsaturated bonds of SM.

**Figure S4** **Confocal microscopy of CY3-labeled P65 protein and DIPA-labeled nucleus.**

**Figure S5** **Correlation diagram of metabolic changes of MH-S cells after co-culture with spores. A** Classification of all metabolites identified. **B** Orthogonal Partial Least Squares Discrimination Analysis (OPLS-DA) diagram of metabolites. **C** OPLS-DA validation model. **D** histogram of differential metabolites. Data are expressed as the mean ± SEM. E The identified metabolites are involved in all pathways.

**Figure S6 Effects of CTRX treatment on the gut microbiota of mice exposed to fungal spores.** (A) Sob index. (B) PCA of fecal metabolites comparing the Fungi and CTRX_F groups; (C) Random Forest Analysis. The larger the mean decrease Gini value, the greater the importance of the dissimilar species. Bubble size varies with species abundance, with red indicating high species abundance and blue indicating low species abundance.

**Figure S7.** **H&E staining, F4/80 staining and PASM staining of the three groups.**

**Figure S8 Lung metabolism and blood metabolism in fungal pneumonia after the recovery of gut microbiota. A** Experimental flow chart. **B** Lung metabolism PCA map. **C** Serum metabolism PCA map. **D** Volcanic map of lung metabolite change. **E** Volcanic map of Serum metabolite change. **F** Lung metabolic TG changes map.


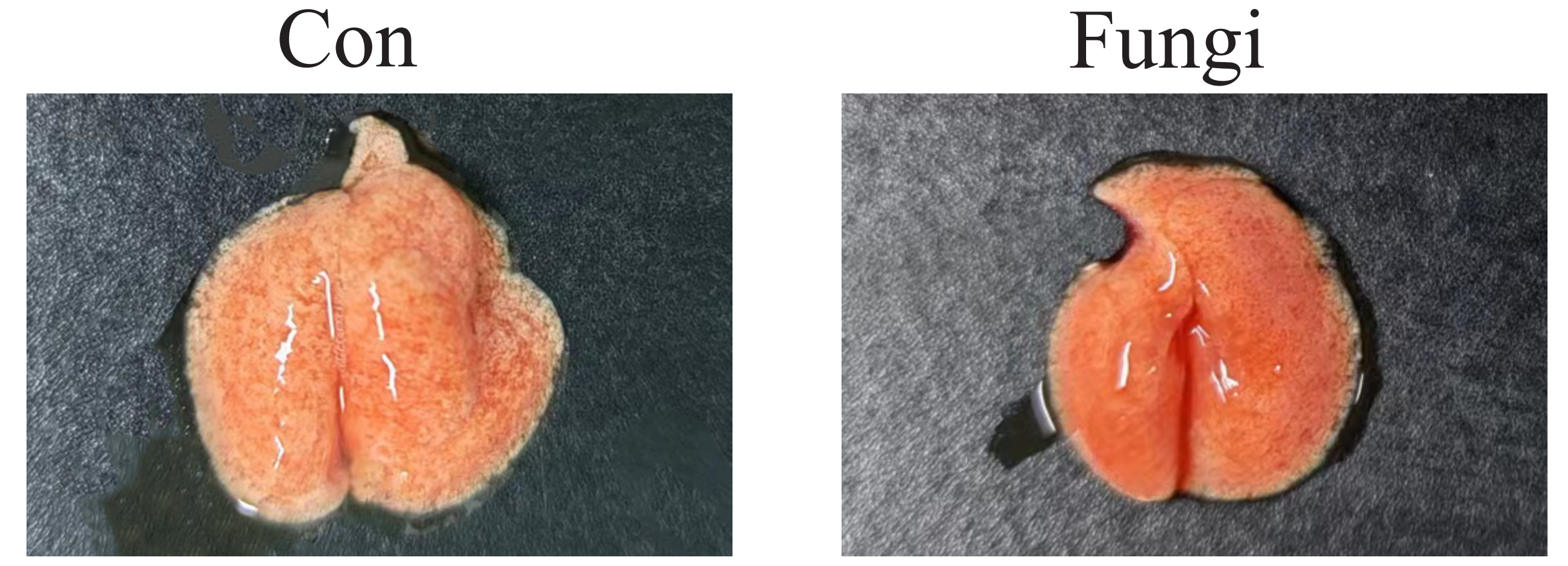


**Figure S1.** Lung tissue images of infected and normal mice revealing hemorrhagic areas caused by F. graminearum infection.

**Figure S2** **Changes of polar and non-polar metabolites in lung.** **A** Nonpolar metabolite species distribution map; **B** Polar metabolite species change distribution map.

**
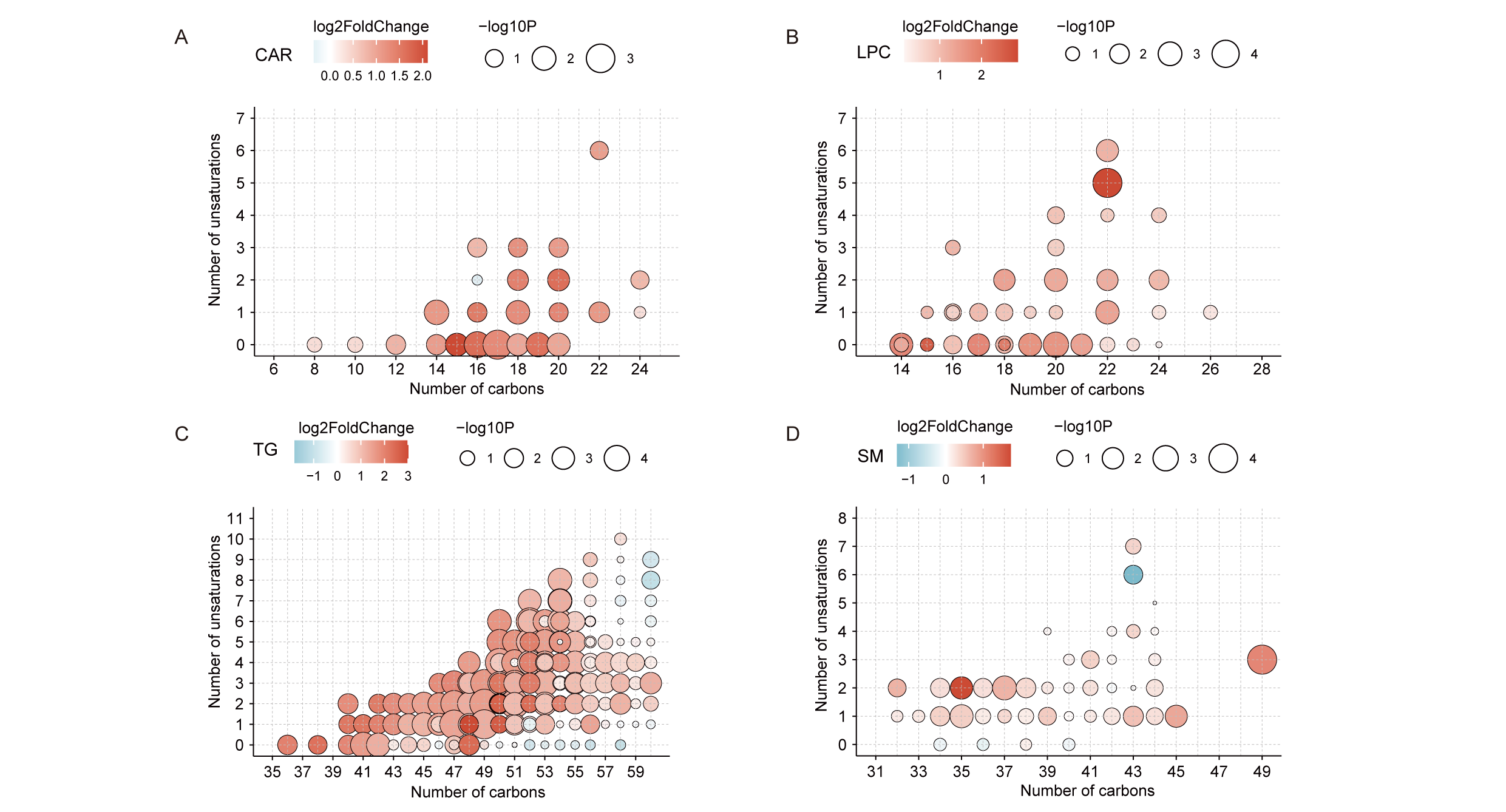
**

**Figure S3** **The number of carbon atoms and unsaturated bonds of CAR, LPC, TG and SM.** **A** The number of carbon atoms and the number of unsaturated bonds of CAR. **B** The number of carbon atoms and the number of unsaturated bonds of LPC. **C** The number of carbon atoms and the number of unsaturated bonds of TG. **D** The number of carbon atoms and the number of unsaturated bonds of SM.

**
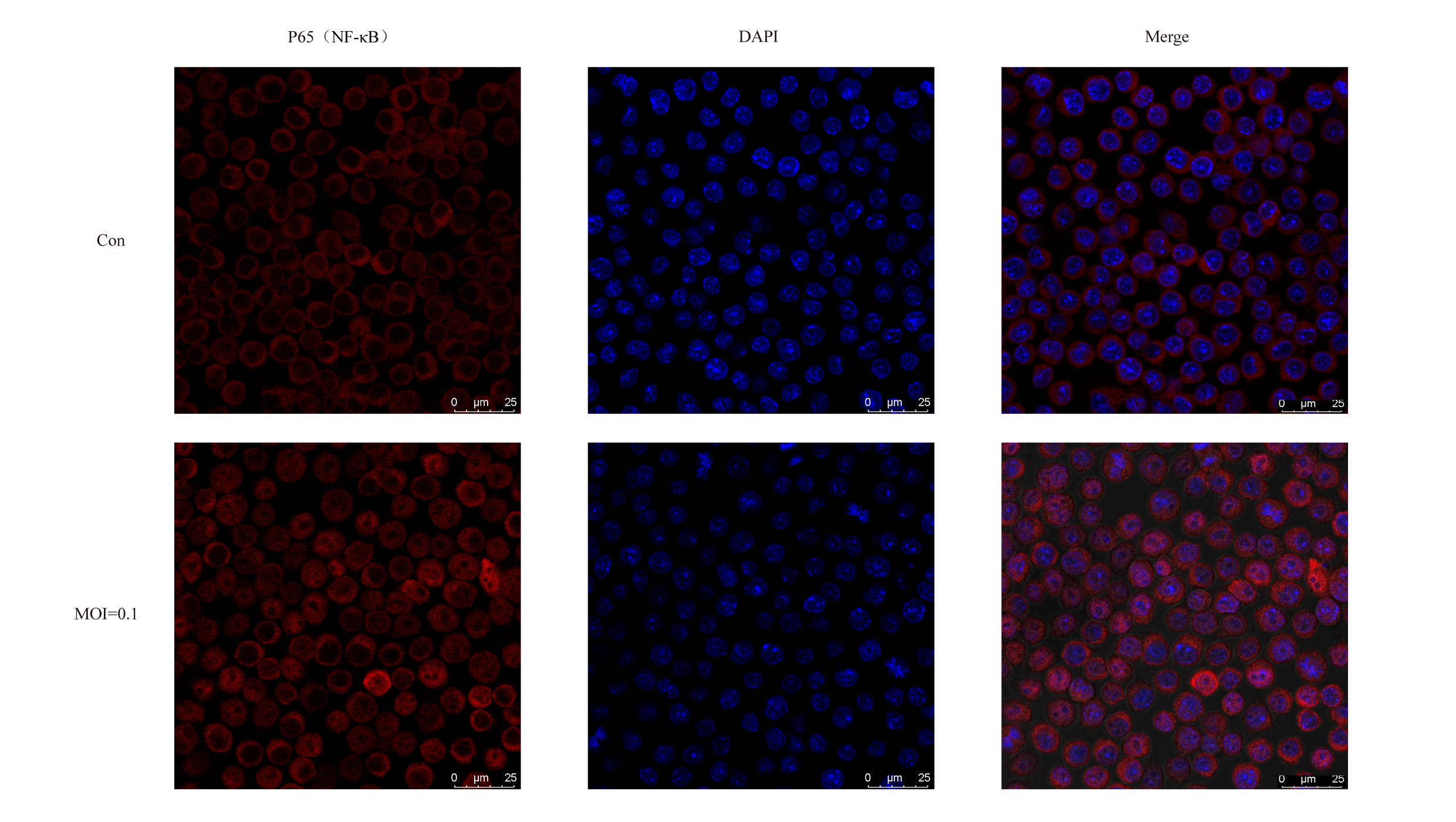
**

**Figure S4** **Confocal microscopy of CY3-labeled P65 protein and DIPA-labeled nucleus.**

**
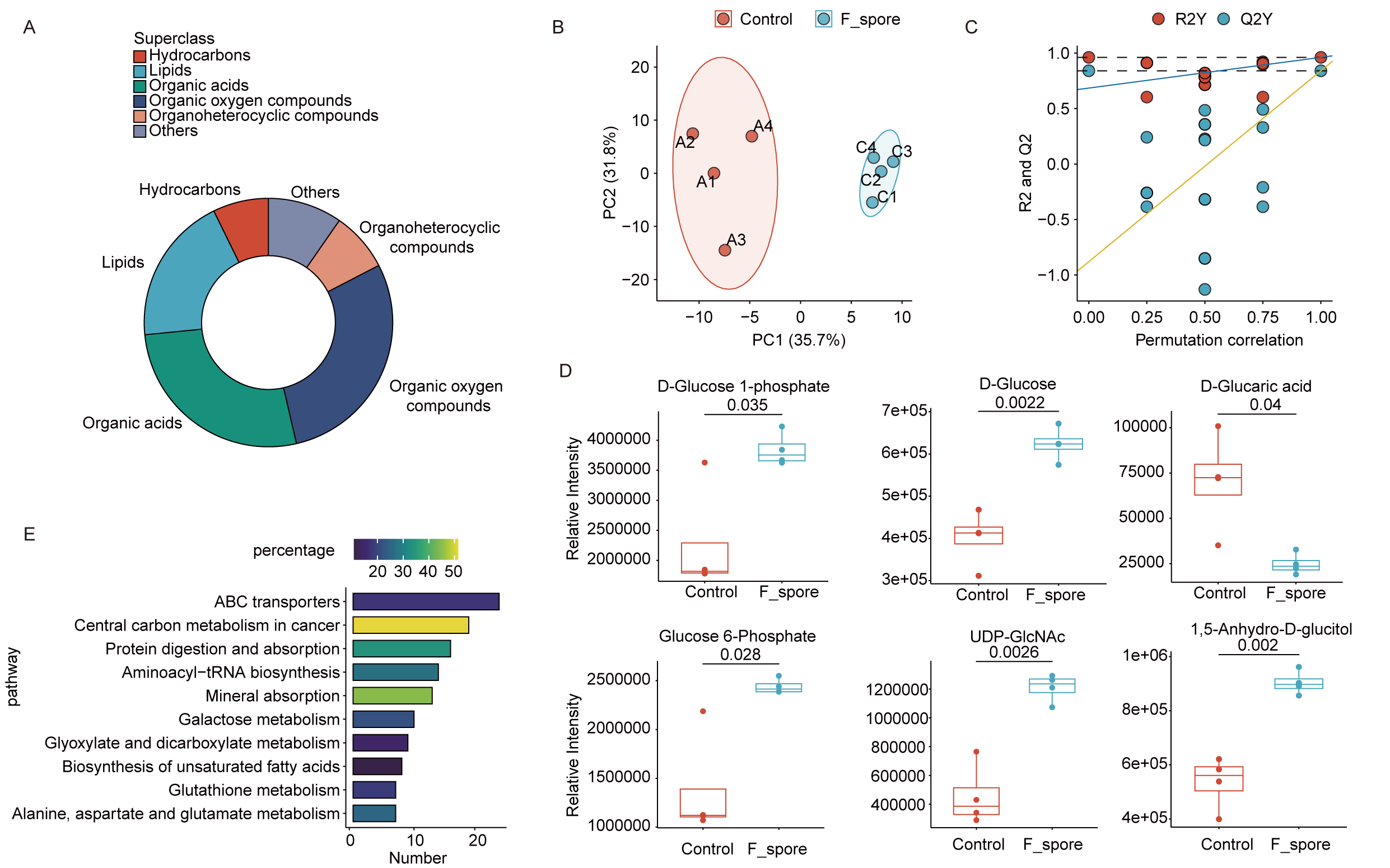
**

**Figure S5** **Correlation diagram of metabolic changes of MH-S cells after co-culture with spores. A** Classification of all metabolites identified. **B** Orthogonal Partial Least Squares Discrimination Analysis (OPLS-DA) diagram of metabolites. **C** OPLS-DA validation model. **D** histogram of differential metabolites. Data are expressed as the mean ± SEM. E The identified metabolites are involved in all pathways.


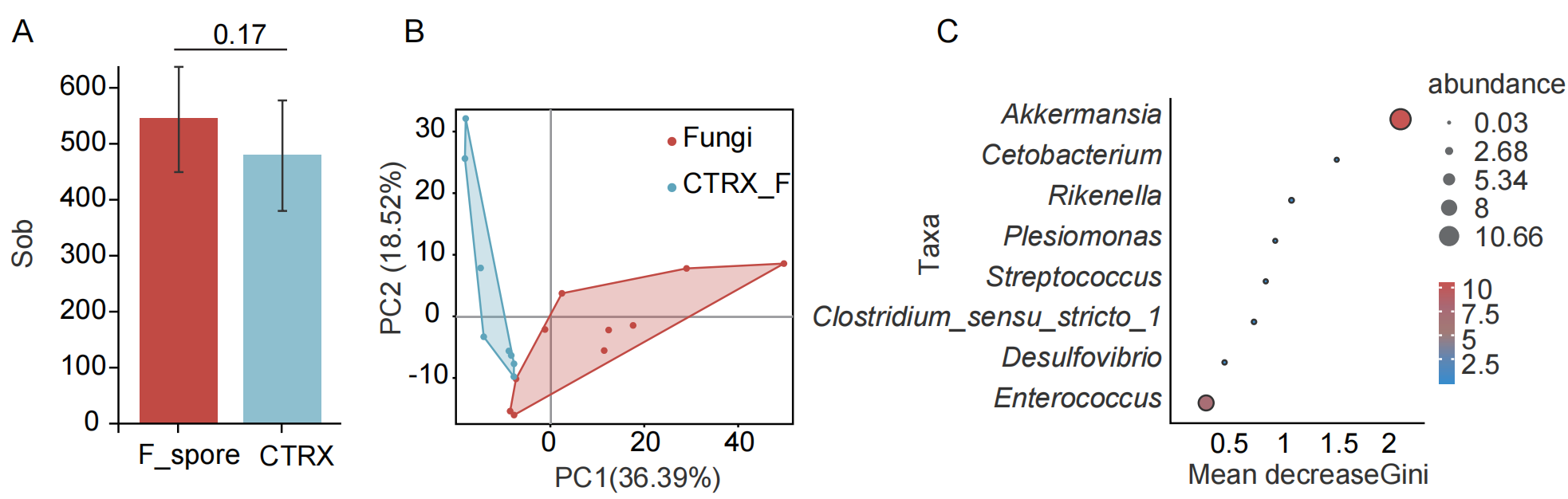


**Figure S6 Effects of CTRX treatment on the gut microbiota of mice exposed to fungal spores.** (A) Sob index. (B) PCA of fecal metabolites comparing the Fungi and CTRX_F groups; (C) Random Forest Analysis. The larger the mean decrease Gini value, the greater the importance of the dissimilar species. Bubble size varies with species abundance, with red indicating high species abundance and blue indicating low species abundance.

**
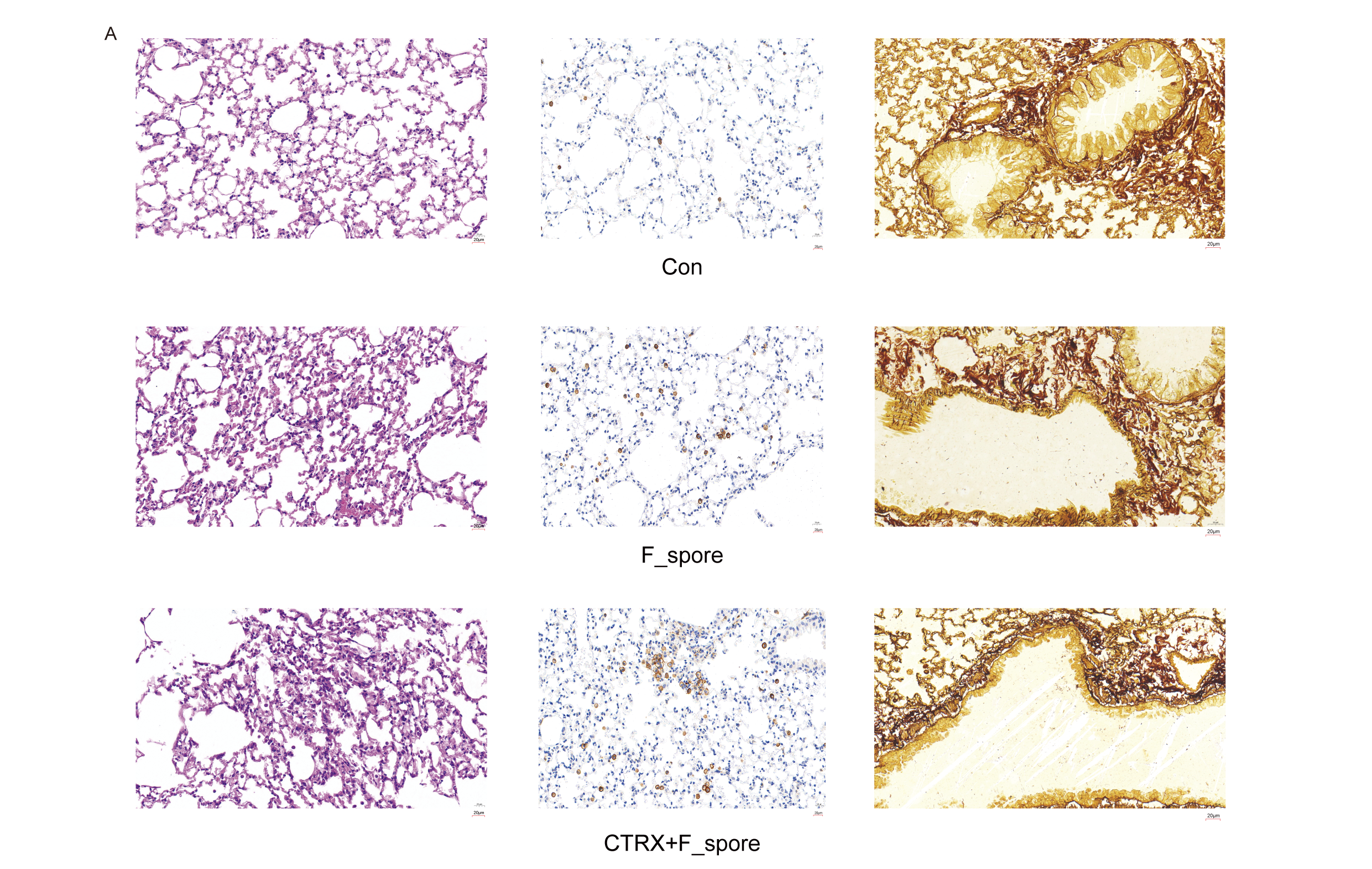
**

**Figure S7 H&E staining, F4/80 staining and PASM staining of the three groups.**


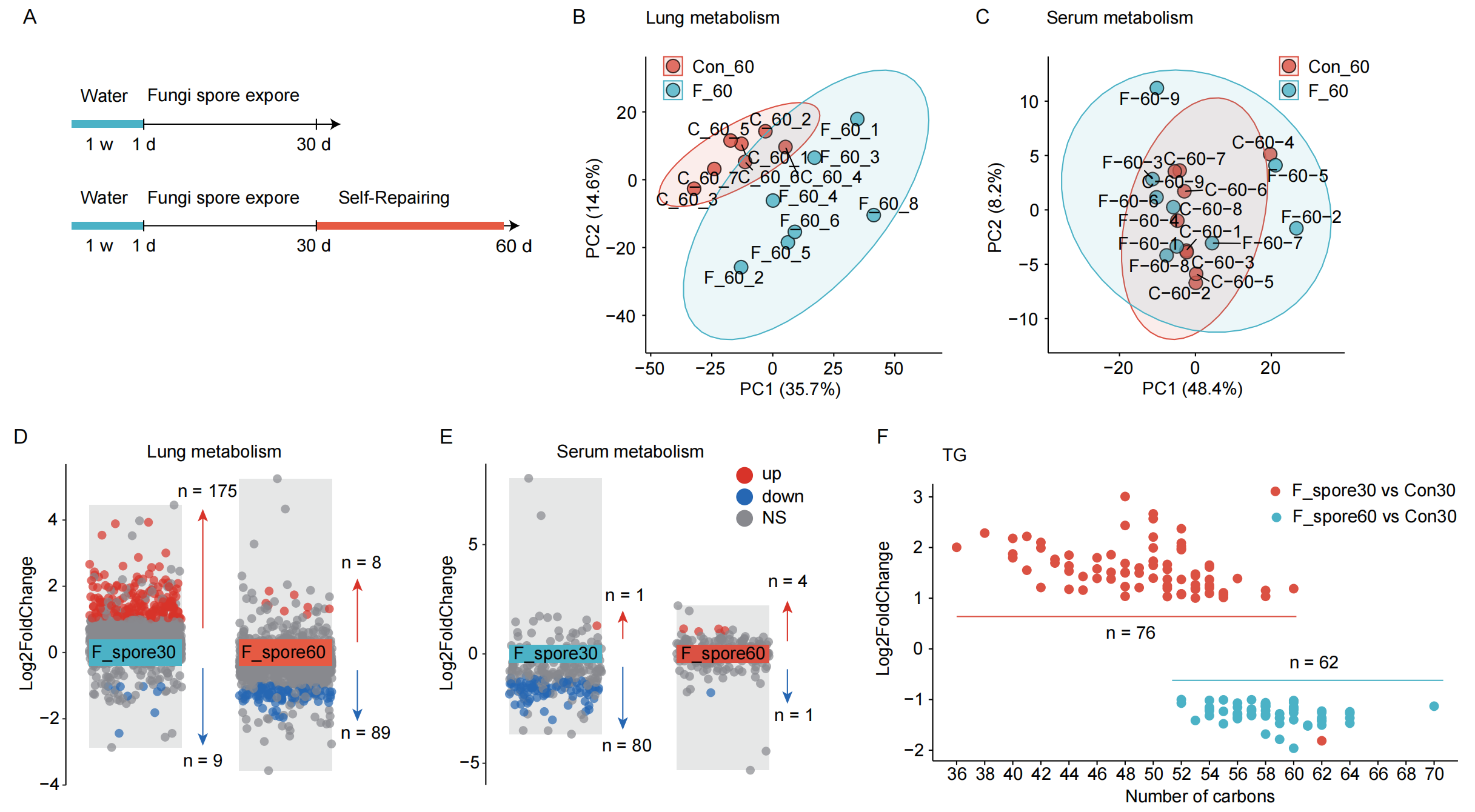


**Figure S8 Lung metabolism and blood metabolism in fungal pneumonia after the recovery of gut microbiota. A** Experimental flow chart. **B** Lung metabolism PCA map. **C** Serum metabolism PCA map. **D** Volcanic map of lung metabolite change. **E** Volcanic map of Serum metabolite change. **F** Lung metabolic TG changes map.
